# SAE-MOGWO-PRS: A probability ranking strategy-based grey wolf optimizer for spatio-temporal adaptive collaborative logistics network optimization

**DOI:** 10.1371/journal.pone.0362456

**Published:** 2026-11-13

**Authors:** Yitong Liu, Tianyu Li, Hong Zhang

**Affiliations:** 1 School of Economics and Management, Tianjin Vocational University, Tianjin, China; 2 School of Management Engineering, Tianjin Modern Vocational Technology College, Tianjin, China; Istinye University: Istinye Universitesi, TURKEY

## Abstract

The spatio-Temporal adaptive collaborative logistics network optimization problems investigated in this paper involve multiple objectives. Most evolutionary multi-objective optimization algorithms, however, do not account for the heterogeneity in evaluation times. Instead, they perform high-fidelity evaluations on all objectives for promising population positions identified by their associated surrogate-assisted optimization algorithms in the solution space. While this approach provides complete and error-free solution evaluations, it does not yield the most computationally efficient procedure. To address this limitation, we propose a novel Surrogate-assisted Evolutionary Multi-objective Grey Wolf Optimizer with Probabilistic Ranking Strategy (SAE-MOGWO-PRS). This algorithm is designed to determine which individuals and objective functions within the population should undergo high-fidelity evaluation, thereby enhancing their ability to generate non-dominated solutions with improved convergence and diversity in the Multi-objective Grey Wolf Optimizer. To validate the efficacy of the proposed SAE-MOGWO-PRS algorithm, we compared it against four state-of-the-art surrogate-assisted multi-objective optimization algorithms, including those that specifically handle heterogeneous evaluation times. The comparative analysis on four test problems demonstrates the superior performance of the proposed probabilistic ranking strategy-based algorithm, thereby confirming the effectiveness of the SAE-MOGWO-PRS method.

## Introduction

In the modern era, logistics systems are increasingly complex, involving dynamic, multi-dimensional networks that span vast geographical areas and operate under varying time constraints [1]. The challenge of optimizing such systems has grown significantly, particularly when considering the integration of spatial and temporal factors in collaborative logistics networks [2]. As supply chains become more interconnected and demand forecasting becomes more intricate, the need for efficient optimization techniques to ensure cost-effectiveness, time sensitivity, and adaptability has never been more pressing.

Spatio-temporal adaptive collaborative logistics networks (ST-CLN) refer to logistics systems that must consider not only the geographic locations of various nodes but also the temporal aspects, such as time windows for deliveries, transportation schedules, and inventory management [3]. These networks need to be optimized for various objectives, including minimizing transportation costs, storage costs, and the penalties incurred due to unmet demand.

In practical applications, these function evaluations typically stem from expensive computational simulations, exemplified by areas such as Proportional Integral Derivative (PID) control [4], heavy-haul transportation management [5], and solar panel parameter identification [6]. Each of these real-world scenarios demands varying amounts of time to evaluate solutions, leading to prohibitive computational costs when hundreds or even tens of thousands of high-fidelity evaluations are required during an optimization run [7]. Furthermore, when tackling problems characterized by conflicting multiple objectives, where each candidate solution must be assessed against every optimization objective, surrogate-assisted optimization has emerged as a powerful approach to mitigate this computational burden [8]. surrogate-assisted models iteratively approximate the expensive objective function evaluation process, thereby enabling efficient and computationally more cost-effective optimization.

Surrogate-assisted models typically involve acquiring high-fidelity candidate solution evaluations either a priori in a single batch or progressively in multiple batches to facilitate the optimization process [8]. While this describes the general framework for single-objective optimization, when extending to multi-objective optimization (MOO), surrogate-assisted MOO models commonly employ space-filling or Latin Hypercube Sampling (LHS) methods to obtain initial high-fidelity evaluations across the entire search space, subsequently constructing the first surrogate models for the objective functions [9,10]. These initial models are then utilized by the optimization algorithm for multiple iterations until a criterion for evaluating candidate solutions with the high-fidelity function is met [11]. However, newly evaluated high-fidelity candidate solutions are subsequently integrated as infill samples to update and refine the surrogate model [12]. It is crucial to acknowledge that surrogate models built on a limited number of infill samples often exhibit substantial prediction errors [13]. Consequently, this necessitates the continuous assessment of new infill samples by the high-fidelity model, which are then used for the iterative improvement of the surrogate model. This progressive (or sequential) surrogate modeling approach represents one of the most advanced methodologies in the field [14].

In this paper, we specifically focus on a class of optimization problems where objective functions exhibit heterogeneous evaluation times. This necessitates a sophisticated scheme to determine which specific objective function for a given proxy-evaluated solution warrants a potentially high-fidelity evaluation. The key innovation of the SAE-MOGWO-PRS algorithm lies in its ability to avoid requiring high-fidelity candidate solution evaluations for all objective functions for every individual in the population, instead allowing for surrogate-based evaluations where appropriate. This process inherently aligns with the principles of multi-fidelity optimization [15].

This heterogeneous evaluation process demands a more complex proxy evaluation management scheme compared to those traditionally employed in surrogate-assisted multi-objective optimization methods. To address the distinct management requirements arising from this heterogeneity, this paper proposes three specific characteristics for each partially surrogate-evaluated solution:

- The relative evaluation time of the surrogate-evaluated objective.
- The prediction error of the objective value estimated by the current surrogate model.
- The selection probability of the candidate solution derived from its non-dominance comparison.

To quantify these three characteristics, we propose a sub-indicator for each, which are then unified to generate a single high-fidelity selection indicator for each objective function of every population member. This high-fidelity selection indicator will serve as the sole criterion for determining which objective function evaluations of candidate solutions require high-fidelity assessment, thereby maintaining the applicability of the entire surrogate-assisted framework to multi-objective optimization problems with two or more objectives.

Numerous studies have explored methods for optimizing problems with heterogeneously expensive objective functions, where the evaluation of different objective functions requires varying computational times. Allmendinger and Knowles [16] proposed an approach to address objective value delays caused by expensive objective functions, introducing three strategies to mitigate these delays. Chugh et al. [17] introduced the HK-RVEA method for tackling bi-objective optimization problems with uneven delays; however, the HK-RVEA method is not applicable when the number of objectives extends to three or more. Thomann and Eichfelder [18] proposed a multi-objective heterogeneous trust-region algorithm that is not based on evolutionary methods. For cases with similar optimization objectives, Wang et al. [19] presented a transfer learning-based method. While most of the aforementioned literature primarily focuses on bi-objective optimization problems, Blank et al. [20] proposed a method for handling multi-objective, multi-constrained heterogeneous function evaluation times. Furthermore, Wang et al. [21] introduced a method to mitigate search bias in multi-objective heterogeneous problems, classifying all objectives into two groups (expensive and cheap) based on a delay ratio threshold; however, this approach does not explicitly consider the heterogeneity within each objective. Vazirani et al. exemplify the application of multi-fidelity Bayesian optimization in the context of high-energy physics, specifically for designing double shell capsules with molybdenum and tungsten inner shells. Their work demonstrates how coupling low- and high-fidelity simulations can effectively explore complex design spaces to identify optimal configurations, highlighting the utility of MFO in physics-based optimization problems [22,23]. Similarly, in aerospace engineering, Nikolaou et al. employ a multi-fidelity surrogate-assisted framework to optimize aircraft wing designs. Their approach enhances aerodynamic performance by balancing the computational cost of low-fidelity models with the accuracy of high-fidelity simulations, thereby improving the lift-to-drag ratio efficiently during early design stages. This underscores MFO’s capacity to accelerate design processes while maintaining solution quality [24]. Liu et al. optimize labyrinth seal designs in aero-engines. Their work demonstrates that multi-fidelity strategies can effectively enhance energy performance by reducing the reliance on expensive high-fidelity evaluations, thus enabling more efficient design iterations [25].

Existing research predominantly focuses on bi-objective problems with heterogeneous objectives, and a comprehensive and general framework for handling heterogeneous objectives with varying evaluation times remains elusive. Extending previous preliminary research [8], we propose a general framework for surrogate-assisted multi-objective optimization that considers three key factors: the relative evaluation time of each objective, the prediction uncertainty of the surrogate model, and the probability advantage guided by reference vectors.

## Literature review

### Grey Wolf Optimizer

The Grey Wolf Optimizer (GWO), a metaheuristic algorithm inspired by grey wolf social hierarchy and hunting behaviour, is valued for its strong convergence and relative simplicity. Recent research focuses on overcoming its limitations, particularly premature convergence and imbalance between exploration and exploitation, through sophisticated enhancements and hybridizations, while expanding its application scope.

Significant efforts target enhancing GWO’s core performance. Multi-strategy approaches are prominent, such as introducing Tent chaos mapping for better initialization and nonlinear convergence factors for balancing exploration-exploitation [26]. Others propose combining ReliefF-based initialization with fitness-weighted position updates and hybridizing GWO with Differential Evolution (DE) and Levy-flight strategies specifically for high-dimensional feature selection [27]. Incorporating flexible crossover and mutation strategies adaptively (GWOC/M) has proven effective for cluster task scheduling in cloud computing [28]. Multi-population strategies combined with dimension learning and Laplace mutation (DLMDGWO) further improve population diversity and global search capability [29]. For binary optimization problems like feature selection, novel binary GWO variants using dynamic position update mechanisms (binDGWO) have been developed [30].

GWO excels in optimizing complex models, particularly in machine learning. It effectively tunes hyperparameters for deep learning architectures, such as optimizing Bi-LSTM networks for diagnosing neuromuscular disorders using EMG signals [31] and enhancing CNN performance for crop disease/pest identification, where a reinforcement learning-based dual-mode GWO was introduced [32]. GWO also optimizes predictive model performance, like integrating with LightGBM and decomposition techniques (CEEMDAN) for accurate influenza trend forecasting [33]. Furthermore, GWO hybrids, particularly LSTM-GWO, significantly outperform standalone models in predicting drilling fluid filtration critical for wellbore stability [34].

GWO’s versatility extends to solving complex real-world problems. It has been applied to multi-objective distributed hybrid flowshop scheduling incorporating reinforcement learning (MOGWO-RL) for mass personalized manufacturing paradigms [35]. In energy systems planning, GWO integrated with EnergyPLAN software optimizes pathways towards carbon neutrality, considering electric vehicle and hydrogen integration for Portugal’s 2050 targets [36]. GWO also contributes to hardware design, enabling compact, high-performance neural processors for acoustic echo cancellation when combined with LMS algorithms [37].

### Logistics networks

Recent research on logistics network optimization increasingly addresses uncertainty, sustainability, and multi-objective trade-offs in complex supply chains. A seminal contribution is Kumar and Mishra’s neutrosophic mixed integer linear programming (NMILP) model, which employs triangular neutrosophic numbers to handle uncertainties in demand, costs, emissions, and delivery times. This approach enables joint optimization of facility location, production allocation, and multimodal transport while balancing cost, carbon, and delay objectives via α, β, γ -cuts [38]. Complementing this, Shi et al. integrate traffic congestion uncertainty and time-varying networks into cold chain logistics, using entropy mechanisms and metaheuristics to optimize joint distribution routes [39].

Customer-centric models are emerging as critical frameworks. Zhang et al. pioneer a fourth-party logistics (4PL) network design that incorporates customer satisfaction-sensitive demand and bounded rationality, solved via Q-learning hyper-heuristics [40]. Similarly, Soria Arguello and García optimize cross-docking operations by synchronizing merchandise location, ergonomics, and distribution costs, highlighting human factors in network design [41]. For demand surge scenarios, Jiang et al. propose chance-constrained capacity planning using a greedy cell-and-bound algorithm to maintain service levels [42].

Sustainability-driven optimization is now ubiquitous. Niu et al. evaluate multi-distribution logistics networks via reliability-based expected performance metrics, linking operational robustness to cost-capacity trade-offs [43]. Wang et al. focus on rural “last-mile” logistics, designing a three-tier green network with fuel-electric mixed fleets under carbon regulations [44]. Reverse logistics networks also advance: He et al. optimize waste battery recycling with technology-tiered facilities [45], while Tosarkani et al. deploy data-driven fuzzy optimization for plastic container recovery networks [46]. Furthermore, various heuristic approaches have been developed to tackle complex optimization tasks in biological and network systems. For instance, advanced evolutionary frameworks have demonstrated effectiveness in modeling complex biological sequences and biological networking [47–49]. Although these methods offer robust search capabilities, they often assume uniform evaluation costs across objectives. Our proposed SAE-MOGWO-PRS builds upon these heuristic principles while explicitly addressing the challenges posed by evaluation time heterogeneity [50].

### Proposed SAE-MOGWO-PRS method

The proposed SAE-MOGWO-PRS method comprises five distinct steps. The overarching process of the SAE-MOGWO-PRS method will be outlined in Algorithm 1, followed by a detailed description of the operations involved in each of these five steps. To enhance the clarity of the proposed algorithm, the key notations and variables used in SAE-MOGWO-PRS are summarized in Table 1.

**Table 1:** Nomenclature and key variables used in SAE-MOGWO-PRS.

Symbol	Description
*N*	Population size
*M*	Number of objective functions
*T*	Maximum number of evaluation cycles
*t*	Current iteration or evaluation cycle
$P_{t,N}$	Population of size *N* at the *t*-th cycle
*Q*_(*t* + 1), *N*_	Offspring population generated by the surrogate-assisted model
$f_{m}(s)$	High-fidelity evaluation of individual *s* for the *m*-th objective
${\tilde{f}}_{m}(s)$	Surrogate-predicted value of individual *s* for the *m*-th objective
$\sigma_{s}^{m}$	Prediction uncertainty (standard deviation) of the surrogate model
$P_{s}^{m}$	Probability of non-deterioration for individual *s* on objective *m*
${\tilde{ET}}^{m}$	Normalized average evaluation time of the *m*-th objective
$\rho_{s}^{m}$	High-fidelity selection (HFS) index for individual *s* on objective *m*
α	Adaptive time-weighting factor
η	Uncertainty control parameter
${\vec{X}}_{\alpha },{\vec{X}}_{\beta },{\vec{X}}_{\delta }$	Positions of the first, second, and third best wolves
*w*	The frequency threshold parameter controlling the execution of the PRS.

**Algorithm 1 Surrogate-assisted Evolutionary Multi-objective Optimization Grey Wolf Optimizer Based on Probability Ranking Strategy (SAE-MOGWO-PRS)**

1: **Input:** $ET_{m}$ (m=1,...,M), *N*, *T*_max_, *N*_SA_, η, $t_{S}$, *E*, $W=\{W_{1},...,W_{N}\}$, *w*, *W*_stag_, $\epsilon_{HV}$, $\delta_{a}$, $S_{\max}$, *a*_reset_

2: **Output:** Final non-dominated solution set

3: Initialize T←0, t←0, s←0

4: $P_{\text{SA}}\leftarrow \text{LHS}(N_{\text{SA}})$

5: **for** m=1,...,M **do**

6:  Evaluate $f_{m}(x_{s})$ for $s=1,...,N_{\text{SA}}$ (high-fidelity)

7:  Set HF flags for all *s* in *P*_SA_ for objective *m*

8:  $T\leftarrow T+ET_{m}\times N_{\text{SA}}$; $A_{m}\leftarrow P_{\text{SA}}$

9:  $K_{m}\leftarrow \text{Surrogate validate}(A_{m},f_{m}(A_{m}),E)$

10:  $SM_{m}\leftarrow \text{Surrogate fit}(A_{m},f_{m}(A_{m}),K_{m})$

11: **end for**

12: $\tau \leftarrow N\sum_{m=1}^{M}ET_{m}$

13: $P_{t\times t_{S}}\leftarrow \text{PRS\_survival}(P_{\text{SA}})$ ▷ size *N*

14: **while** $T\leq T_{\max}-\tau$ **do**

15:  $Q_{(t+1)\times t_{S}}\leftarrow \text{SA}(P_{t\times t_{S}},\;t_{S},\;SM)$ ▷ Surrogate evaluation only; HF flags remain **False**

16:  $R\leftarrow \text{Eliminate duplicates}(P_{t\times t_{S}}\cup Q_{(t+1)\times t_{S}})$

17:  *SM*.evaluate(*R*)

18:  $\gamma_{k}\leftarrow \text{Association}(R,W_{k})$ for k=1,...,N

19:  $P_{(t+1)\times t_{S}}\leftarrow \text{Select}(R,A,N,T,\eta ,\gamma_{k},ET)$

20:  **for** m=1,...,M **do**

21:   $K_{m}\leftarrow \text{Surrogate validate}(A_{m},f_{m}(A_{m}),E)$

22:   $SM_{m}\leftarrow \text{Surrogate fit}(A_{m},f_{m}(A_{m}),K_{m})$

23:  **end for**

24:  **Adaptive *a*:** $a_{\text{lin}}\leftarrow 2-2t/T_{\max}$; compute ${\overset{―}{\Delta HV}}_{k}$ over *W*_stag_

25:  **if** ${\overset{―}{\Delta HV}}_{k}<\epsilon_{HV}$ **then** s←s+1; $a\leftarrow a_{\text{lin}}+\delta_{a}\;\cdot \;\min(s/S_{\max},\;1)$

26:   **if** $s>S_{\max}$ **then** $a\leftarrow a_{\text{reset}}$; s←0

27:   **end if**

28:  **else** s←0; $a\leftarrow a_{\text{lin}}$

29:  **end if**

30:  Update *A*, *C* parameters of MOGWO using *a*

31:  **MOGWO Search:** update wolf positions via ${\vec{X}}_{\alpha },{\vec{X}}_{\beta },{\vec{X}}_{\delta }$

32:  **if** *mod*(*t*, *w*) = 0 **then** ▷ PRS: selective high-fidelity evaluation

33:   Compute $P_{s}^{m}$ and $\sigma_{s}^{m}$; compute HFS index $\rho_{s}^{m}$ (Eq. 18)

34:   Select top-ranked (individual, objective) pairs based on $\rho_{s}^{m}$

35:   **Set HF flag** ← **True** for selected pairs **only**

36:   Perform HF evaluation on selected pairs; update *A*; advance *T*

37:  **end if**

38:  **if** *mod*(*t*, *w*_adapt_) = 0 **then** ▷ Reference vector adaptation

39:   Identify inactive vectors *W*_inactive_ with zero solutions over last window

40:   Identify crowded vectors *W*_crowd_ exceeding threshold *N*_crowd_

41:   Reassign each $W\in W_{\text{inactive}}$ by bisecting top-ranked $W\in W_{\text{crowd}}$

42:  **end if**

43:  t←t+1

44: **end while**

45: Evaluate final population with high-fidelity evaluations

### Initialization

The proposed SAE-MOGWO-PRS method commences with an initial set of $N_{SA}$ samples generated via Latin Hypercube Sampling (LHS). These initial high-fidelity candidate solutions are then evaluated, and a separate surrogate model is constructed for each objective function. The evaluated content for each optimization objective is stored in an elite archive $A_{m}$. The total computational time τ expended on evaluating the initial experimental design is updated within the evaluation budget as $\tau =N_{SA}\sum_{m=1}^{M}E\times T^{m}$. This formulation provides a conservative upper bound on the consumed budget; in practice, parallel evaluation reduces the actual wall-clock time, and the budget tracking during the evolutionary loop adopts a wall-clock-based accounting as detailed in Section. As Tian et al. [8] have demonstrated the advantages of the Kriging method for constructing surrogate models—specifically its ability to predict objective values and provide uncertainty information as a measure of modeling error—we have chosen to employ Kriging for building the surrogate models in the SAE-MOGWO-PRS method. For the construction of these surrogate models, the $N_{SA}$ data points are divided into training and validation sets using three-fold cross-validation. The best-performing model is then selected from six different regressors based on Mean Absolute Error (MAE). The chosen model is subsequently utilized for the next round of surrogate model updates within the selected multi-objective grey wolf algorithm (MOGWO).

### Multi-objective Grey Wolf Algorithm (MOGWO)

The Grey Wolf Optimizer (GWO) algorithm was proposed by Mirjalili and Lewis in 2014 [51]. The social hierarchy and hunting behavior of grey wolf packs served as the primary inspiration for the development of the GWO algorithm. For modeling purposes, the GWO algorithm abstracts the wolf pack’s social hierarchy, designating the current best solution in the population as the α wolf, and the second and third best solutions as the β and δ wolves, respectively. The remaining wolves in the population are considered ω wolves. In the GWO algorithm, the hunting process is primarily guided by the α, β, and δ wolves, while the ω wolves predominantly follow the activities of these leader wolves. Beyond this social leadership structure, the GWO algorithm primarily simulates the encircling behavior of grey wolf packs during hunting based on Eq.s (1) and (2).


$$\begin{array}{c} \vec{D}=\left|\vec{C}\times \vec{X_{p}}(t)-\vec{X}(t)\right| \end{array}$$ (1)

$$\begin{array}{c} \vec{X}(t+1)=\vec{X_{p}}(t)-\vec{A}\times \vec{D} \end{array}$$ (2)

where, *t* denotes the current iteration number of the GWO algorithm. $\vec{A}$ and $\vec{C}$ are coefficient vectors associated with the encircling hunting behavior. $\vec{X_{p}}$ represents the position vector of the prey, while $\vec{X}$ denotes the position vector of a grey wolf (individual). The Eq.s (3) and (4) provide the calculation process for these coefficient vectors.


$$\begin{array}{c} \vec{A}=2\times \vec{a}\times \vec{rand_{1}}-\vec{a} \end{array}$$ (3)

$$\begin{array}{c} \vec{C}=2\times \vec{rand_{2}} \end{array}$$ (4)

where, throughout the entire iterative process of the GWO algorithm, the value of the element $\vec{a}$ linearly decreases from 2 to 0, while $\vec{rand_{1}}$ and $\vec{rand_{2}}$ are random vectors with components uniformly distributed within the range [0, 1].

The GWO algorithm leverages its simulated social hierarchy and encircling mechanism to find the optimal solution for optimization problems. The algorithm maintains the three best solutions obtained thus far, which then guide the position updates of the other search agents (including the ω wolves). During the optimization process, each search agent continuously updates its position using Eq.s (5) to (11) to simulate the search and explore promising regions of the search space:


$$\begin{array}{c} \vec{D_{\alpha }}=\left|\vec{C_{1}}\times \vec{X_{\alpha }}-\vec{X}\right| \end{array}$$ (5)

$$\begin{array}{c} \vec{D_{\beta }}=\left|\vec{C_{2}}\times \vec{X_{\beta }}-\vec{X}\right| \end{array}$$ (6)

$$\begin{array}{c} \vec{D_{\delta }}=\left|\vec{C_{3}}\times \vec{X_{\delta }}-\vec{X}\right| \end{array}$$ (7)

$$\begin{array}{c} \vec{X_{1}}=\vec{X_{\alpha }}-\vec{A_{1}}\times \vec{D_{\alpha }} \end{array}$$ (8)

$$\begin{array}{c} \vec{X_{2}}=\vec{X_{\beta }}-\vec{A_{2}}\times \vec{D_{\beta }} \end{array}$$ (9)

$$\begin{array}{c} \vec{X_{3}}=\vec{X_{\delta }}-\vec{A_{3}}\times \vec{D_{\delta }} \end{array}$$ (10)

$$\begin{array}{c} \vec{X}(t+1)=\frac{\vec{X_{1}}+\vec{X_{2}}+\vec{X_{3}}}{3} \end{array}$$ (11)

The exploration behavior of the GWO algorithm is governed by the values of $\vec{A}$, which are determined by random values greater than 1 or less than −1. To adapt GWO for multi-objective optimization, two main components are incorporated in this paper. The first component is an archive, primarily responsible for storing the non-dominated Pareto optimal solutions obtained thus far. The second component is a leader selection strategy, which selects the α, β, and δ wolves from the archive component to serve as leaders for the MOGWO algorithm’s search process, based on criteria consistent with the GWO algorithm’s principles.

The archive functions as a straightforward storage unit. An archive management controller governs its operations, particularly when new candidate solutions need to be added or when the archive reaches its capacity. The following rules dictate its management:

- If a new candidate solution is to be added to a full archive and it is dominated by at least one solution already in the archive, then the new candidate solution is not admitted.
- If a new candidate solution dominates one or more solutions currently residing in the archive, then the dominated solutions are removed, and the new candidate solution is added to the archive.
- If the new candidate solution and existing archive members are mutually non-dominated, the new candidate solution is added to the archive.
- If the archive reaches its full capacity, a target-space network mechanism is employed to reorganize the archive’s contents. This mechanism identifies regions with the highest crowding density among candidate solutions in the objective space and removes redundant solutions. Subsequently, the new candidate solution is added to the least crowded position. This operation enhances the diversity of the algorithm’s Pareto front solutions.

The leader selection mechanism is a crucial component when extending single-objective optimization to multi-objective problems. In single-objective optimization, the selection of α, β, and δ wolves solely relies on the magnitude of the objective function value. However, in a multi-objective search space, the magnitude of objective function values cannot directly determine the superiority or inferiority of solutions. The leader selection mechanism addresses this challenge by first identifying regions with the least crowding density in the objective space and then selecting their non-dominated solutions to serve as the α, β, and δ wolves. This selection process is implemented via a roulette wheel selection method, where the selection probability for each hypercube is given by Eq. (12).


$$\begin{array}{c} P_{i}=\frac{c}{N_{i}} \end{array}$$ (12)

where, *c* is a constant greater than 1, and *N* represents the number of Pareto optimal solutions obtained in the *i*-th segment, as evident in Eq. (12). Hypercubes with lower crowding density have a higher probability of yielding new leaders. Consequently, as the number of solutions within a hypercube decreases, the probability of selecting that hypercube for leader nomination increases.

The standard GWO algorithm decays the parameter $\vec{a}$ linearly from 2 to 0 (Eq. 3), implicitly assuming a uniform exploration-to-exploitation transition across all iterations. However, in heterogeneous multi-objective optimization of spatio-temporal logistics networks, the convergence landscape varies significantly across problems due to differing evaluation-time asymmetries and rugged objective surfaces. A rigid linear schedule may cause premature convergence (insufficient exploration) or delayed convergence (insufficient exploitation). To address this, we introduce a feedback-driven adaptive mechanism that modulates $\vec{a}$ based on the dynamics of the elite archive $A_{m}$. Specifically, we monitor the Hypervolume (*HV*) improvement rate over a sliding window of *W* surrogate update cycles. Let $\Delta HV_{k}=HV(A_{m}^{\left(k\right)})-HV(A_{m}^{\left(k-1\right)})$ denote the *HV* increment at the *k*-th surrogate update. The average improvement rate over the window is:


$$\begin{array}{c} {\overset{―}{\Delta HV}}_{k}=\frac{1}{W}\sum_{i=k-W+1}^{k}\Delta HV_{i} \end{array}$$ (13)

When ${\overset{―}{\Delta HV}}_{k}<\epsilon_{HV}$—indicating convergence stagnation—a stagnation counter *s* is incremented. The parameter $\vec{a}$ is then adaptively adjusted as:


$$\begin{array}{c} a(t)=\left\{ \begin{array}{cc} a_{\text{linear}}(t)+\delta_{a}\cdot \frac{s}{S_{\max}}, & 0<s\leq S_{\max}\\ a_{\text{reset}}, & s>S_{\max} \end{array}\right. \end{array}$$ (14)

where $a_{\text{linear}}(t)=2-2t/T_{\max}$ is the standard linear decay, $\delta_{a}$ is the exploration boost step, $S_{\max}$ is the stagnation tolerance threshold, and $a_{\text{reset}}\in [1.5,2.0]$ is a reset value that re-injects exploration when stagnation persists beyond tolerance. When no stagnation is detected (*s* = 0), $\vec{a}$ reverts to the standard linear decay, preserving the GWO’s original exploitation efficiency. This adaptive strategy allows SAE-MOGWO-PRS to dynamically rebalance exploration and exploitation according to the actual convergence progress on each specific problem instance.

### Surrogate-assisted MOGWO Generations

During the $t_{S}$ iterations of the *t*-th surrogate update cycle wi*t*hin the surrogate-assisted MOGWO algorithm, we use the parent population $P_{t\times t_{S}}$ as the initial population to run the MOGWO algorithm. In this paper, we set the value of $t_{S}$ to 30. Upon completion of these $t_{S}$ iterations, an offspring population $Q_{(t+1)\times t_{S}}$ containing *N* individuals is generated. At this stage, we begin with a parent population $P_{t\times t_{S}}$, where individuals have undergone partial high-fidelity evaluation. The resulting offspring population $Q_{(t+1)\times t_{S}}$ is then entirely evaluated using the current surrogate models.

Two subsequent tasks are then performed. The first task involves merging the parent and offspring populations, forming $R_{(t+1)\times t_{S}}=P_{t\times t_{S}}\cup Q_{(t+1)\times t_{S}}$, from which a population of size *N* is selected to proceed to the next generation’s iteration. The second task is to determine which members and objectives require subsequent high-fidelity evaluation. This is crucial for updating the current surrogate models, allowing the optimization to progress towards the optimal region, both by identifying representative solutions and by improving the surrogate approximation. Regarding this second task, we first detail the calculation process for the high-fidelity selection measure $\rho_{s}^{m}$ for each objective function *m* of every population member *s* within the combined population $R_{t\times t_{S}}$. This measure will then be utilized in the aforementioned first task.

In practice, Kriging models can suffer from numerical instability or degradation when the accumulated high-fidelity data points are unevenly distributed or when the correlation matrix becomes ill-conditioned due to closely spaced training samples — both of which are likely to occur as the population converges toward the Pareto front. To safeguard against degraded surrogate predictions corrupting the PRS selection, we introduce a lightweight health check after each surrogate model update. For each objective *m*, the current surrogate model $SM_{m}^{\left(t\right)}$ is validated on a held-out subset of the archive $A_{m}$. If its Mean Absolute Error (MAE) exceeds a deterioration threshold $\theta_{\text{MAE}}$ relative to the best MAE recorded in previous cycles, the model is flagged as unreliable. The algorithm then applies a two-tier fallback. In the first tier, the surrogate model from the most recent healthy cycle is reused, under the assumption that the objective landscape has not shifted sufficiently to invalidate the older model within a single surrogate update interval. In the second tier — invoked if no healthy model exists or if the deterioration persists for more than *S*_surr_ consecutive cycles — the algorithm temporarily suspends surrogate-based evaluation for the affected objective. Instead, it forces high-fidelity evaluation for that objective in the next PRS cycle, thereby injecting fresh data to restore model quality. Once the surrogate model recovers and its MAE falls below the threshold, normal surrogate-assisted operation resumes. This mechanism ensures that PRS selections are never guided by numerically unstable or diverged surrogate predictions, while incurring minimal additional computational overhead since the health check uses only the already-available validation data.

In practical implementation, high-fidelity evaluations for different (individual, objective) pairs are submitted as independent asynchronous tasks and executed in parallel whenever computational resources permit. When the PRS strategy determines that a specific objective function of a given individual requires high-fidelity evaluation, the corresponding evaluation task is dispatched immediately and the algorithm proceeds without waiting for its completion. The surrogate models continue to serve as proxy evaluators for guiding the MOGWO search in the intervening period. The total elapsed wall-clock time *T* is advanced only when an evaluation task actually completes: if multiple evaluations run concurrently, *T* is incremented by the maximum elapsed duration among the concurrently running tasks rather than by their sum. This asynchronous model ensures that CPU cycles are not wasted in idle states — fast evaluations can be submitted, completed, and integrated into the surrogate models while slow evaluations are still in progress. Consequently, the surrogate models are updated incrementally as new high-fidelity results become available, rather than only at discrete synchronization points. The surrogate update frequency parameter $t_{S}$ thus governs the interval at which the MOGWO population is refreshed using the latest surrogate models, not a hard synchronization barrier that requires all pending evaluations to complete. This design guarantees that the computational budget utilization reflects realistic parallel execution and that the algorithm maintains forward progress regardless of objective-wise evaluation-time heterogeneity. When an objective is flagged for forced high-fidelity evaluation due to persistent surrogate degradation, its HFS index receives a priority boost to ensure that fresh data are collected in the immediate next PRS cycle, thereby restoring the surrogate model’s reliability without disrupting the overall optimization flow.

### High-fidelity selection metric computation

To select which objective function *m* for each population member s from the combined population $R_{(t+1)\times t_{S}}$ requires high-fidelity evaluation, we compute a high-fidelity selection indicator based on three aspects:

- The probability that the region around the current member s will not worsen, denoted as $P_{s}^{m}$.
- The relative expected computation time of individuals within the combined population $R_{(t+1)\times t_{S}}$, denoted as $\overset{⌢}{ET^{m}}$.
- The uncertainty of the surrogate model, $\sigma_{s}^{m}$.

The first aspect considers the quality of candidate solution *s* for objective function *m* within its neighborhood. The second aspect represents the relative evaluation time of objective function *m*. The third aspect, conversely, indicates the quality of the surrogate model in approximating the objective function’s evaluation. It is noteworthy that since the high-fidelity evaluation scheme permits only one applicable reference vector at a time, the preservation of diversity is also implicitly ensured.

First, to calculate $P_{s}^{m}$, we determine the average probability $PD_{s}^{m}$ that candidate solution s is worse than all other members associated with the same reference vector $W_{k}$, while also considering the uncertainty $\sigma_{s}^{m}$ in the predicted surrogate values. To minimize the m-th objective, the probability $P_{sy}^{m}$ that member s (with predicted objective value $\mu_{s}^{m}$ and uncertainty $\sigma_{s}^{m}$ is worse than another related solution y (with prediction $\mu_{y}^{m}$ and uncertainty $\sigma_{y}^{m}$) can be expressed using the convolution of two normal distributions, as shown below:


$$\begin{array}{c} P_{sy}^{m}=\text{Prob}\left[f_{m}(s)>f_{m}(y)\right]=\frac{1+\text{erf}(-z)}{2} \end{array}$$ (15)

where, $z=\frac{\left(0-(\mu_{s}^{m}-\mu_{y}^{m})\right)}{\sqrt{2\times ((\sigma_{s}^{m}{)}^{2}+(\sigma_{y}^{m}{)}^{2})}}$ and erf() denotes the Gaussian error function.


$$\begin{array}{c} PD_{s}^{m}=\frac{\sum_{i\in \pi (s),i\neq s}P_{si}^{m}}{\left|\pi (s)-1\right|} \end{array}$$ (16)

For instance, as illustrated in Fig 1, the average probability $PD_{P2}^{m}$ for solution *P*2 in objectives *f*1 (*m* = 1) and *f*2 (*m* = 2) is calculated as: $PD_{P2}^{1}=\frac{P_{23}^{1}+P_{24}^{1}}{3-1}$ and $PD_{P2}^{2}=\frac{P_{23}^{2}+P_{24}^{2}}{3-1}$. This assumes three inter-related members exist along the reference direction *W*2. After computing the $PD_{s}^{m}$ metric for each population $R_{(t+1)\times t_{S}}$ across all objective functions, we can then determine the probability that individual s is not worse than its related members, expressed as $P_{s}^{m}=1-PD_{s}^{m}$. Intuitively, assuming no other influencing factors, an objective with a larger $P_{s}^{m}$ value should be subjected to high-fidelity evaluation more frequently.

**Fig 1:**
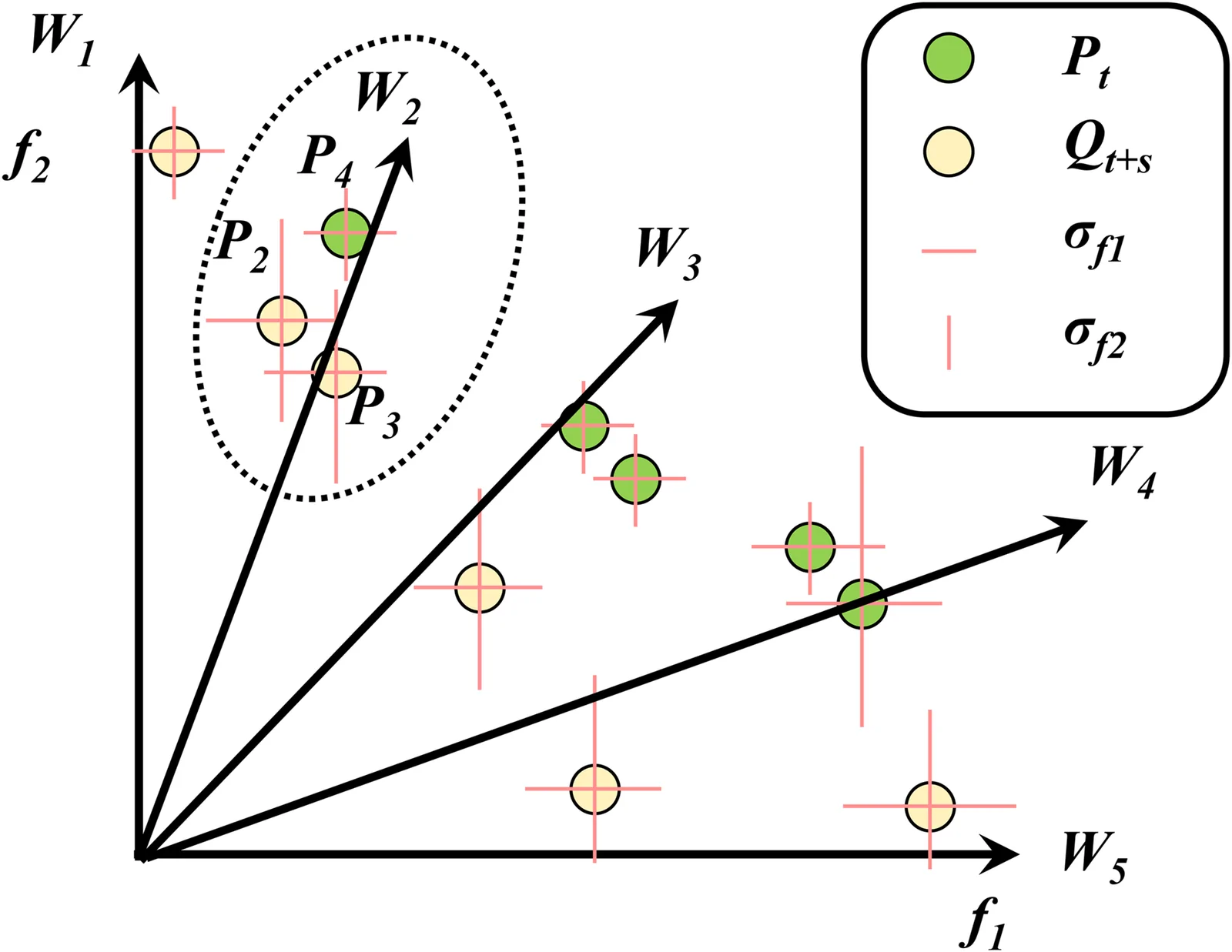
Probabilistic domination computation for reference vector.

Next, we consider the impact of the evaluation time for each objective. Given the average evaluation time for the *m*-th objective ($ET^{m}$), we normalize it by the maximum evaluation time among all objectives to compute the HFS indicator, as follows:


$$\begin{array}{c} {\tilde{ET}}^{m}=\frac{ET^{m}}{\text{max}(ET^{1},ET^{2},\cdots ,ET^{M})} \end{array}$$ (17)

This normalization ensures that each $\tilde{ET^{m}}$ is less than or equal to 1, thereby providing a relative estimation of the heterogeneous evaluation times. Intuitively, in the early stages of the optimization run, a larger $\tilde{ET^{m}}$ might warrant a lower probability of being assigned a high-fidelity evaluation. However, as iterations progress, objectives with larger $\tilde{ET^{m}}$ values must undergo high-fidelity evaluation to accurately locate approximate optimal solutions.

Finally, we consider the surrogate prediction error for the *m*-th objective ($\sigma_{s}^{m}$), which is normalized by the range of $f_{m}$ values within the population. Evidently, a higher ($\sigma_{s}^{m}$) implies a larger prediction error and, consequently, a greater probability for its high-fidelity evaluation, thereby enabling more accurate surrogate estimations.

We now combine these three factors to construct the following High-Fidelity Selection (HFS) measure $\rho_{s}^{m}$ for solution *s* and objective *m* [52,53]:


$$\begin{array}{c} \rho_{s}^{m}=P_{s}^{m}\times \left(1+\alpha \cdot {\tilde{ET}}^{m}\right)\times \left(1+{\left(\frac{\sigma_{s}^{m}}{\left|\Delta f_{m}\right|}\right)}^{\frac{1}{\eta }}\right) \end{array}$$ (18)

Where ${\tilde{ET}}^{m}$ denotes the normalized average evaluation time for the *m*-th objective function. It should be noted that *ET* is a single compounded notation for evaluation time rather than a product of two variables *E* and *T*. The term α∈[−1,1] is the adaptive time-weighting factor, which coordinates the selection priority between low-cost and high-cost objectives at different evolutionary stages. The term $\left|\Delta f_{m}\right|=\tilde{z_{m}^{nad}}-\tilde{z_{m}^{*}}$ serves as a normalization factor for the uncertainty term of the *m*-th objective function, where $\tilde{z_{m}^{nad}}$ and $\tilde{z_{m}^{*}}$ are the estimated nadir and ideal points, respectively, obtained from the current population, similar to MOGWO. The parameter η>0 acts as a control parameter, emphasizing the importance of uncertainty in deriving the overall measure. The effect of the term ${\tilde{ET}}^{m}$ is introduced by the control parameter α∈[−1,1], which is further investigated in the parameter study of this research.

A well-known limitation of fixed uniformly distributed reference vectors is their poor coverage when the Pareto front is irregular or disconnected — a situation commonly encountered in spatio-temporal logistics optimization where certain trade-off regions are physically infeasible. To ensure that the neighborhood-based non-dominance comparison in PRS remains meaningful throughout the optimization, we introduce a periodic reference vector adaptation mechanism. Every *w*_adapt_ surrogate update cycles, reference vectors that have accumulated zero associated non-dominated solutions over the preceding window are identified and flagged as inactive. Concurrently, reference vectors whose associated solution count exceeds a crowding threshold *N*_crowd_ are identified as over-subscribed. Each inactive vector is then reassigned by bisecting one of the most crowded vectors in objective space, with the new direction anchored toward the centroid of archived solutions within that crowded region. This ensures that the adaptive reference vectors gradually converge toward the true geometry of the Pareto front while maintaining a total of *N* vectors. As a result, the neighborhood density estimates used in computing the non-deterioration probability $P_{s}^{m}$ remain robust even when the underlying Pareto front deviates substantially from the uniform simplex.

### Survival selection for high-fidelity evaluations

After computing the $\rho_{s}^{m}$ measure for the entire $R_{(t+1)\times t_{S}}$ population across all objectives, we transform the ρ measures into a 1-D vector, stacking them for all solutions across all objectives relative to their reference vectors. To select members and their corresponding objectives for high-fidelity evaluation, we iterate along each reference vector direction $W_{k}$. For each $W_{k}$, we select the solution $\overset{―}{s}$ and its corresponding objective $\overset{―}{m}$ that yields the maximum $\rho_{s}^{m}$ value among all related members associated with that reference vector. This solution $\overset{―}{s}$ is then chosen for high-fidelity evaluation if it has not been previously evaluated at high-fidelity in $P_{t\times t_{S}}$ or during the current selection process. Subsequently, the selected member $\overset{―}{s}$ is stored in $S_{t\times t_{S}}$. We repeat this process until exactly *N* members have been selected into $S_{t\times t_{S}}$. It is important to note that some selected members might be chosen for high-fidelity evaluation across multiple objective functions, while others may be selected for only a single objective function. Furthermore, certain objectives of some selected members might have already undergone high-fidelity evaluation. The *N* selected solutions, comprising a mix of high-fidelity and surrogate-evaluated individuals, constitute $P_{(t+1)\times t_{S}}$. The entire process is illustrated in Fig 2.

**Fig 2:**
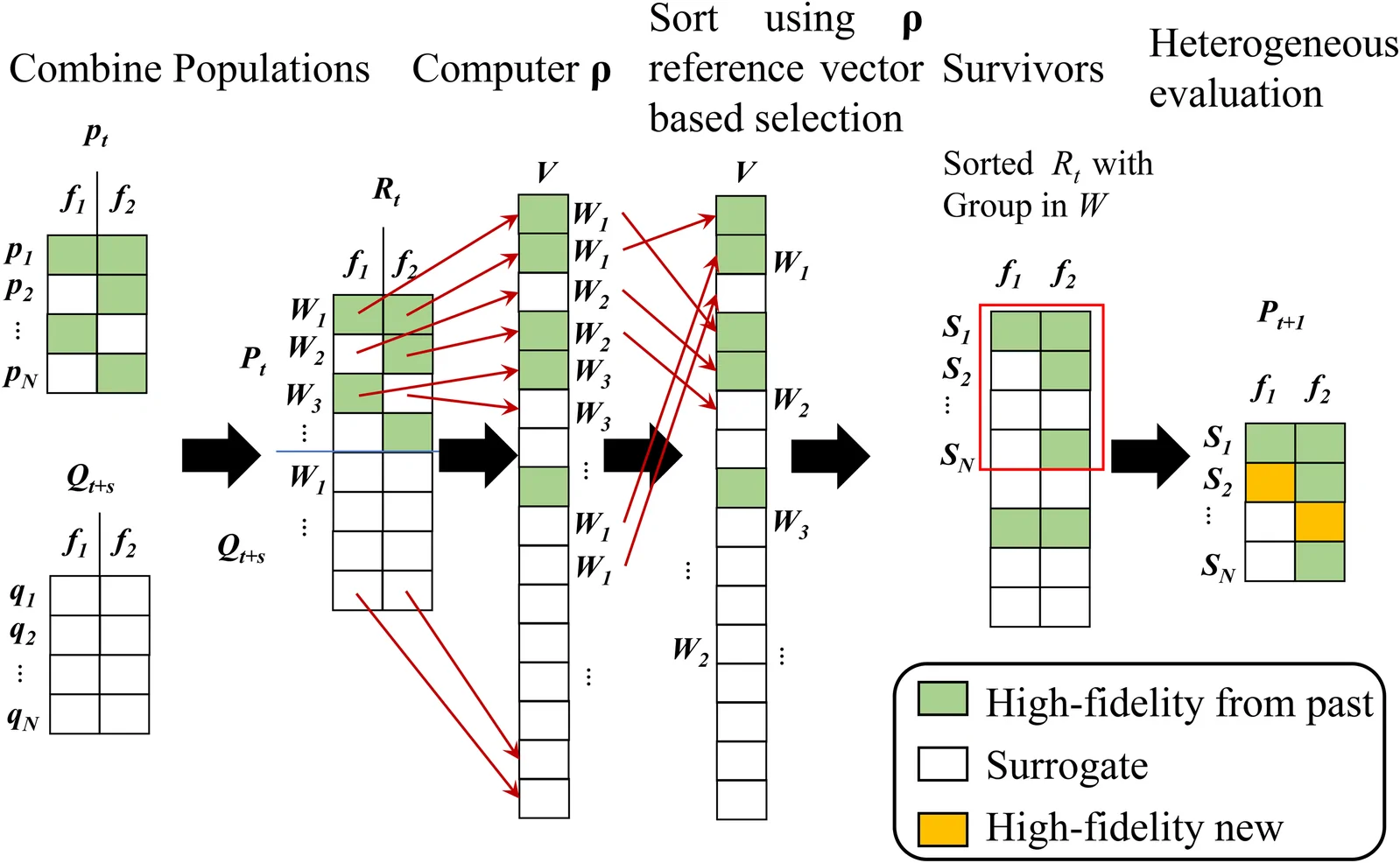
Survival selection to construct.

### Adaptive use of parameter α

In the early stages of the optimization process, solutions are typically far from the optimal region, rendering precise evaluations unnecessary. Therefore, it is reasonable to initially employ a negative α value, which emphasizes objective functions with lower computational costs. However, as iterations progress, potentially promising solutions discovered through lower-cost objective functions may necessitate a positive α value to facilitate their high-fidelity evaluation for more computationally expensive objectives. We propose the following standard linear mapping update rule for the α parameter:


$$\begin{array}{c} \alpha =2\times \left(\frac{t}{T_{max}}\right)-1 \end{array}$$ (19)

The dynamic adjustment of the control parameter α, as defined in Eq. (17), is designed to balance the search efficiency and solution precision across different evolutionary stages. Here, *t* denotes the current i*t*eration and $T_{max}$ represents the maximum number of iterations. During the early stage of optimization (when *t* is small), α yields a negative value. This configuration effectively reduces the HFS index for objectives with high evaluation cos*t*s, thereby guiding the algorithm to prioritize’fast’ objectives to rapidly explore the solution space. As the evolution progresses to the later stage, α gradually increases and transitions to a positive value, correctly mapping the iteration progression into the interval [−1,1]. This shift ensures that the algorithm allocates more computational budget to high-fidelity evaluations of time-consuming objectives, which is essential for refining the final Pareto front and ensuring the accuracy of the non-dominated solutions. In the initial phase, our focus is more on evaluating lower-cost objectives. In later stages, to obtain more accurate evaluations, we prioritize time-consuming objectives. It should be noted that objectives that are frequently subjected to high-fidelity evaluation due to their lower cost may exhibit small surrogate errors.

## Spatio-temporal adaptive collaborative logistics network optimization model

### Problem description

In the context of modern logistics, efficient management and optimization of the logistics network have become increasingly important due to the growing demand for timely deliveries, cost reductions, and sustainability goals. The Spatio-Temporal Adaptive Collaborative Logistics Network Optimization Problem (ST-CLNOP) involves optimizing the operations of a logistics network while considering both spatial (location-based) and temporal (time-based) aspects of logistics processes. The problem aims to coordinate logistics resources across multiple stages (e.g., transportation, warehousing, inventory) with dynamic adaptability to real-time information and disruptions in a collaborative network structure.

Given that logistics networks often involve multiple participants (e.g., suppliers, distributors, retailers), and a variety of operational constraints, the challenge is to optimize logistics flow in a way that maximizes efficiency while minimizing costs, delivery time, and resource wastage, under both planned and unforeseen circumstances.

(1) **Variables**
  $x_{ijt}$: The amount of goods transported from node *i* to node *j* at time *t*, where i,j∈{1,2,...,N} epresent the locations in the logistics network, and t∈{1,2,...,T} represents the discrete time periods.
  $y_{it}$: The quantity of goods stored at node *i* at time *t*.
(2) **Parameters**
  $c_{ij}$: The cost per unit of transporting goods from node *i* to node *j*.
  $d_{it}$: The demand at node *i* at time *t*.
  $s_{it}$: The supply at node *i* at time *t*.
  $w_{ijt}$: The transportation capacity between node *i* to node *j* at time *t*.
  $p_{i}$: The cost of storing one unit of goods at node *i*.
  $r_{t}$: The time-dependent capacity of the transportation network

### Objective function

In the context of the Spatio-Temporal Adaptive Collaborative Logistics Network Optimization Problem (ST-CLNOP), the optimization process seeks to balance several competing objectives to ensure that the logistics network operates efficiently, effectively, and cost-effectively. The objective function of the ST-CLNOP can be expressed as the minimization of the total logistics cost, which includes transportation costs, storage costs, and penalty costs due to unfulfilled demands.

#### (1) Minimization of transportation costs.

The transportation cost represents the expense incurred when moving goods between different nodes within the logistics network across both space (location-based) and time (temporal considerations). This cost is a fundamental objective because transportation typically represents a significant portion of the overall logistics cost. Mathematically, the transportation cost can be formulated as shown in Eq 20.


$$\begin{array}{c} F1=\sum_{i=1}^{N}\sum_{j=1}^{N}\sum_{t=1}^{T}c_{ij}x_{ijt} \end{array}$$ (20)

#### (2) Minimization of storage costs.

The storage cost refers to the expense associated with holding goods at various nodes (e.g., warehouses, distribution centers) in the logistics network. Storage is a critical aspect of supply chain management, and excessive or inefficient storage can lead to unnecessary costs, such as warehousing fees, inventory holding costs, and potential inventory obsolescence. Mathematically, the storage cost can be represented as shown in Eq 21.


$$\begin{array}{c} F2=\sum_{i=1}^{N}\sum_{t=1}^{T}p_{i}y_{ij} \end{array}$$ (21)

#### (3) Minimization of penalty costs due to unmet demand.

The penalty costs arise when demand is not satisfied within the specified time frame. In a real-world logistics system, unmet demand can result from transportation delays, insufficient stock, or bottlenecks within the network. These penalties serve as a way to quantify the financial repercussions of failing to meet customer expectations. Mathematically, the penalty cost for unmet demand can be expressed as shown in Eq 22.


$$\begin{array}{c} F3=\sum_{i=1}^{N}\sum_{t=1}^{T}\left(d_{it}-\sum_{j=1}^{N}x_{ijt}\right) \end{array}$$ (22)

### Constraints

The ST-CLNOP involves several constraints that need to be satisfied during the optimization process. These constraints ensure that the logistics network operates within the physical, capacity, and time limitations.

#### (1) Demand satisfaction.

The demand satisfaction constraint ensures that the demand at each node *i* is satisfied at each time period *t*, as shown in Eq 23.


$$\begin{array}{c} \sum_{j=1}^{N}x_{ijt}=d_{it},\;\forall i\in \{1,2,...,N\}\&\&\forall t\in \{1,2,...,T\} \end{array}$$ (23)

#### (2) Supply Availability.

The supply availability constraint ensures that the supply at each node *i* is not exceeded in any period of time *t*, as shown in Eq 24.


$$\begin{array}{c} \sum_{j=1}^{N}x_{ijt}\leq s_{it},\;\forall i\in \{1,2,...,N\}\&\&\forall t\in \{1,2,...,T\} \end{array}$$ (24)

#### (3) Transportation capacity.

The transportation capacity constraint ensures that the transportation quantity from node *i* to node *j* does not exceed the available transportation capacity at any time period *t*, as shown in Eq 25.


$$\begin{array}{c} x_{ijt}\leq w_{ijt},\;\forall i,j\in \{1,2,...,N\}\&\&\forall t\in \{1,2,...,T\} \end{array}$$ (25)

#### (4) Storage capacity.

The storage capacity constraint ensures that the storage quantity at each node *i* at any time period *t* does not exceed the storage capacity of node *i*, as shown in Eq 26.


$$\begin{array}{c} y_{it}\leq Capacity_{i},\;\forall i\in \{1,2,...,N\}\&\&\forall t\in \{1,2,...,T\} \end{array}$$ (26)

where $Capacity_{i}$ is the storage capacity of node *i*.

#### (5) Flow Conservation.

The flow conservation constraint ensures that the total amount of goods entering and leaving node *i* is balanced over time, as shown in Eq 27.


$$\begin{array}{c} \sum_{j=1}^{N}x_{ijt}-\sum_{j=1}^{N}x_{jit}=0,\;\forall i,j\in \{1,2,...,N\}\&\&\forall t\in \{1,2,...,T\} \end{array}$$ (27)

#### (6) Time dependency.

The time dependency constraint limits the total transportation volume at each time period *t* to the network’s transportation capacity $r_{t}$, as shown in Eq 28.


$$\begin{array}{c} \sum_{i=1}^{N}\sum_{j=1}^{N}x_{ijt}\leq r_{t},\;\forall t\in \{1,2,...,T\} \end{array}$$ (28)

## Experimental results and cases study

### Benchmark results comparison and analysis

We tested the proposed SAE-MOGWO-PRS method against four competing algorithms: (i) the original MOGWO, where every population member undergoes high-fidelity evaluation for all objectives without using any surrogate model; (ii) DOSA-PIO [8], which optimizes using a surrogate model every $t_{S}$ generations, but all offspring population members of $Q_{t\times t_{S}}$ are subjected to high-fidelity evaluation for all objectives before the surrogate model is updated for the next generation; (iii) ParEGO [54], which employs a generative surrogate-assisted approach but does not handle heterogeneity in objective evaluations; (v) K-RVEA [55], a surrogate-assisted and reference vector-guided evolutionary multi-objective algorithm, also without addressing heterogeneity in objective evaluations. For all test problems, the number of decision variables was set to 10. For problems with three objective functions, the population size (*N*) was set to 50.

The selection of the four comparison algorithms follows a hierarchical validation strategy. MOGWO is chosen to provide a baseline for the core optimizer performance. ParEGO and K-RVEA are included as state-of-the-art surrogate-assisted algorithms to evaluate the efficiency of the proposed PRS strategy in managing high-fidelity evaluation budgets. DOSA-PIO is selected specifically due to its reported adaptability in handling computationally expensive objectives. Collectively, these algorithms represent both classical and recent advancements in the SAEMO field, providing a rigorous benchmark for assessing the ability of SAE-MOGWO-PRS to handle heterogeneous evaluation times.

The total termination time $T_{max}$ was set to 300, and the initial experimental design size $N_{SA}$ was set to 100. As previously mentioned, we assume the estimated total evaluation time for solutions across all objectives to be $t_{total}=1$ unit, and we utilize the relative evaluation time ($ET^{m}$) for each objective. For instance, $ET^{1}=ET^{2}=0.5$ indicates that the average evaluation time for solutions for both objectives is approximately equal. However, *ET*^3^ = 0.33 and *ET*^4^ = 0.67 imply that the average evaluation time of the second objective is almost double that of the first.

It is worth noting that the evaluation-time configurations in Table 2 are designed such that the per-individual total evaluation cost $\sum_{m=1}^{3}ET_{m}$ equals 1.0 across all nine configurations. This intentional normalization ensures that the total number of complete individual evaluations achievable within the fixed time budget $T_{\max}=300$ remains constant for algorithms that evaluate all objectives jointly, namely MOGWO, ParEGO, and K-RVEA. Since these three algorithms treat all objectives symmetrically and do not incorporate any per-objective selectivity in their evaluation strategy, they naturally converge to statistically indistinguishable IGD^+^ values across all ET configurations for each given test problem. The identical values appearing across rows for these algorithms are therefore a direct mathematical consequence of the evaluation budget design, not an artifact of data duplication. In contrast, DOSA-PIO and SAE-MOGWO-PRS exhibit IGD^+^ variation across ET configurations precisely because their heterogeneity management strategies selectively allocate evaluation resources across objectives, making their performance sensitive to the underlying cost distribution. This budget design thus serves a deliberate experimental purpose: it isolates the contribution of heterogeneity management by ensuring that performance differences among ET configurations arise solely from how efficiently each algorithm allocates its high-fidelity evaluation budget across objectives with asymmetric costs.

**Table 2:** Median IGD^+^ value comparison of algorithms for three-objective DTLZ and WFG problems. Note: All nine evaluation-time configurations satisfy $\sum_{m=1}^{3}ET_{m}=1.0$ by design, ensuring that the total per-individual evaluation cost is normalized across rows. Consequently, algorithms that do not differentiate among objectives (MOGWO, ParEGO, and K-RVEA) perform an identical number of complete high-fidelity evaluations under every configuration, and their identical IGD^+^ values across the nine rows for each problem are a direct and expected consequence of this budget normalization, not a table artifact. In contrast, DOSA-PIO and SAE-MOGWO-PRS, which selectively evaluate subsets of objectives based on their respective heterogeneity management strategies, exhibit variation across configurations. Standard deviations and complete per-run statistics are provided in Table 3.

Problem	($ET^{1},ET^{2},ET^{3},$)	MOGWO	DOSA-PIO	ParEGO	K-RVEA	SAE-MOGWO-PRS
DTLZ2	(0.5, 0.33, 0.17)	2.584E-1(-)	4.851E-2(=)	1.212E-1(-)	1.776E-1(-)	4.327E-2
	(0.5, 0.17, 0.33)	2.584E-1(-)	4.851E-2(=)	1.212E-1(-)	1.776E-1(-)	4.464E-2
	(0.5, 0.1, 0.4)	2.584E-1(-)	4.851E-2(-)	1.212E-1(-)	1.776E-1(-)	4.391E-2
	(0.33, 0.33, 0.33)	2.584E-1(-)	4.851E-2(-)	1.212E-1(-)	1.776E-1(-)	4.517E-2
	(0.33, 0.5, 0.17)	2.584E-1(-)	4.851E-2(=)	1.212E-1(-)	1.776E-1(-)	4.366E-2
	(0.33, 0.17, 0.5)	2.584E-1(-)	4.851E-2(-)	1.212E-1(-)	1.776E-1(-)	4.411E-2
	(0.17, 0.5, 0.33)	2.584E-1(-)	4.851E-2(-)	1.212E-1(-)	1.776E-1(-)	4.365E-2
	(0.12, 0.08, 0.8)	2.584E-1(-)	4.851E-2(-)	1.212E-1(-)	1.776E-1(-)	3.744E-2
	(0.08, 0.12, 0.8)	2.584E-1(-)	4.851E-2(=)	1.212E-1(-)	1.776E-1(-)	3.752E-2
DTLZ5	(0.5, 0.33, 0.17)	2.816E-1(-)	3.755E-2(-)	6.633E-2(-)	7.986E-2(-)	1.661E-2
	(0.5, 0.17, 0.33)	2.816E-1(-)	3.755E-2(-)	6.633E-2(-)	7.986E-2(-)	1.637E-2
	(0.5, 0.1, 0.4)	2.816E-1(-)	3.755E-2(-)	6.633E-2(-)	7.986E-2(-)	1.636E-2
	(0.33, 0.33, 0.33)	2.816E-1(-)	3.755E-2(-)	6.633E-2(-)	7.986E-2(-)	1.675E-2
	(0.33, 0.5, 0.17)	2.816E-1(-)	3.755E-2(-)	6.633E-2(-)	7.986E-2(-)	1.732E-2
	(0.33, 0.17, 0.5)	2.816E-1(-)	3.755E-2(-)	6.633E-2(-)	7.986E-2(-)	1.887E-2
	(0.17, 0.5, 0.33)	2.816E-1(-)	3.755E-2(-)	6.633E-2(-)	7.986E-2(-)	2.198E-2
	(0.12, 0.08, 0.8)	2.816E-1(-)	3.755E-2(-)	6.633E-2(-)	7.986E-2(-)	1.905E-2
	(0.08, 0.12, 0.8)	2.816E-1(-)	3.755E-2(-)	6.633E-2(-)	7.986E-2(-)	1.731E-2
DTLZ7	(0.5, 0.33, 0.17)	1.756E + 0(-)	6.767E-1(=)	5.368E-2(-)	4.965E-2(=)	3.869E-2
	(0.5, 0.17, 0.33)	1.756E + 0(-)	6.767E-1(-)	5.368E-2(=)	4.965E-2(-)	3.867E-2
	(0.5, 0.1, 0.4)	1.756E + 0(-)	6.767E-1(-)	5.368E-2(=)	4.965E-2(=)	4.162E-2
	(0.33, 0.33, 0.33)	1.756E + 0(-)	6.767E-1(-)	5.368E-2(-)	4.965E-2(=)	4.076E-2
	(0.33, 0.5, 0.17)	1.756E + 0(-)	6.767E-1(-)	5.368E-2(-)	4.965E-2(=)	3.976E-2
	(0.33, 0.17, 0.5)	1.756E + 0(-)	6.767E-1(-)	5.368E-2(-)	4.965E-2(=)	3.917E-2
	(0.17, 0.5, 0.33)	1.756E + 0(-)	6.767E-1(=)	5.368E-2(=)	4.965E-2(=)	4.281E-2
	(0.12, 0.08, 0.8)	1.756E + 0(-)	6.767E-1(-)	5.368E-2(-)	4.965E-2(=)	3.857E-2
	(0.08, 0.12, 0.8)	1.756E + 0(-)	6.767E-1(=)	5.368E-2(-)	4.965E-2(=)	3.869E-2
WFG3	(0.5, 0.33, 0.17)	2.766E-1(-)	8.788E-2(-)	1.717E-1(-)	1.369E-1(-)	5.378E-2
	(0.5, 0.17, 0.33)	2.766E-1(-)	8.788E-2(-)	1.717E-1(-)	1.369E-1(-)	5.765E-2
	(0.5, 0.1, 0.4)	2.766E-1(-)	8.788E-2(-)	1.717E-1(-)	1.369E-1(-)	5.672E-2
	(0.33, 0.33, 0.33)	2.766E-1(-)	8.788E-2(-)	1.717E-1(-)	1.369E-1(-)	6.075E-2
	(0.33, 0.5, 0.17)	2.766E-1(-)	8.788E-2(-)	1.717E-1(-)	1.369E-1(-)	4.971E-2
	(0.33, 0.17, 0.5)	2.766E-1(-)	8.788E-2(-)	1.717E-1(-)	1.369E-1(-)	5.855E-2
	(0.17, 0.5, 0.33)	2.766E-1(-)	8.788E-2(-)	1.717E-1(-)	1.369E-1(-)	5.856E-2
	(0.12, 0.08, 0.8)	2.766E-1(-)	8.788E-2(-)	1.717E-1(-)	1.369E-1(-)	5.957E-2
	(0.08, 0.12, 0.8)	2.766E-1(-)	8.788E-2(-)	1.717E-1(-)	1.369E-1(-)	5.307E-2
WFG6	(0.5, 0.33, 0.17)	3.127E-1(-)	1.729E-1(=)	1.762E-1(=)	1.808E-1(-)	9.705E-2
	(0.5, 0.17, 0.33)	3.127E-1(-)	1.729E-1(-)	1.762E-1(-)	1.808E-1(-)	1.071E-1
	(0.5, 0.1, 0.4)	3.127E-1(-)	1.729E-1(=)	1.762E-1(=)	1.808E-1(-)	9.527E-2
	(0.33, 0.33, 0.33)	3.127E-1(-)	1.729E-1(=)	1.762E-1(=)	1.808E-1(-)	9.765E-2
	(0.33, 0.5, 0.17)	3.127E-1(-)	1.729E-1(-)	1.762E-1(=)	1.808E-1(-)	9.761E-2
	(0.33, 0.17, 0.5)	3.127E-1(-)	1.729E-1(=)	1.762E-1(-)	1.808E-1(-)	9.732E-2
	(0.17, 0.5, 0.33)	3.127E-1(-)	1.729E-1(-)	1.762E-1(-)	1.808E-1(-)	9.617E-2
	(0.12, 0.08, 0.8)	3.127E-1(-)	1.729E-1(=)	1.762E-1(-)	1.808E-1(-)	9.765E-2
	(0.08, 0.12, 0.8)	3.127E-1(-)	1.729E-1(=)	1.762E-1(-)	1.808E-1(-)	9.325E-2
WFG8	(0.5, 0.33, 0.17)	3.461E-1(-)	2.973E-1(-)	2.867E-1(-)	2.574E-1(-)	1.986E-1
	(0.5, 0.17, 0.33)	3.461E-1(-)	2.973E-1(-)	2.867E-1(-)	2.574E-1(-)	1.982E-1
	(0.5, 0.1, 0.4)	3.461E-1(-)	2.973E-1(-)	2.867E-1(-)	2.574E-1(-)	1.863E-1
	(0.33, 0.33, 0.33)	3.461E-1(-)	2.973E-1(-)	2.867E-1(-)	2.574E-1(-)	1.897E-1
	(0.33, 0.5, 0.17)	3.461E-1(-)	2.973E-1(-)	2.867E-1(-)	2.574E-1(-)	2.062E-1
	(0.33, 0.17, 0.5)	3.461E-1(-)	2.973E-1(-)	2.867E-1(-)	2.574E-1(-)	1.993E-1
	(0.17, 0.5, 0.33)	3.461E-1(-)	2.973E-1(-)	2.867E-1(-)	2.574E-1(-)	1.986E-1
	(0.12, 0.08, 0.8)	3.461E-1(-)	2.973E-1(-)	2.867E-1(-)	2.574E-1(-)	1.878E-1
	(0.08, 0.12, 0.8)	3.461E-1(-)	2.973E-1(-)	2.867E-1(-)	2.574E-1(-)	1.907E-1
+/ = /-		0/0/54	0/13/41	0/7/47	0/8/46	38/16/0

**Table 3:** Complete IGD^+^ statistics (mean ± standard deviation over 20 independent runs) for all algorithms on three-objective DTLZ and WFG problems. The Wilcoxon signed-rank test results (+/-/=) at α=0.05 are reported in parentheses. Note that MOGWO, ParEGO, and K-RVEA do not differentiate among objectives; since all ET configurations satisfy $\sum_{m=1}^{3}ET_{m}=1.0$, their mean IGD^+^ values are statistically indistinguishable across rows. The small but observable variations in standard deviations reflect the independent random seeds used in each 20-run experiment, confirming that each row reports a distinct set of experimental trials.

Problem	($ET^{1},ET^{2},ET^{3}$)	MOGWO	DOSA-PIO	ParEGO	K-RVEA	SAE-MOGWO-PRS
DTLZ2	(0.50, 0.33, 0.17)	2.584E-1 ± 3.2E-3(-)	4.851E-2 ± 2.1E-3(=)	1.212E-1 ± 5.4E-3(-)	1.776E-1 ± 6.8E-3(-)	4.327E-2 ± 1.9E-3
	(0.50, 0.17, 0.33)	2.583E-1 ± 3.5E-3(-)	4.849E-2 ± 2.3E-3(=)	1.211E-1 ± 5.1E-3(-)	1.775E-1 ± 7.1E-3(-)	4.464E-2 ± 2.0E-3
	(0.50, 0.10, 0.40)	2.585E-1 ± 2.9E-3(-)	4.853E-2 ± 1.9E-3(-)	1.213E-1 ± 5.6E-3(-)	1.777E-1 ± 6.5E-3(-)	4.391E-2 ± 1.8E-3
	(0.33, 0.33, 0.33)	2.584E-1 ± 3.4E-3(-)	4.850E-2 ± 2.2E-3(-)	1.212E-1 ± 5.3E-3(-)	1.776E-1 ± 6.9E-3(-)	4.517E-2 ± 2.2E-3
	(0.33, 0.50, 0.17)	2.584E-1 ± 3.1E-3(-)	4.852E-2 ± 2.0E-3(=)	1.212E-1 ± 5.5E-3(-)	1.776E-1 ± 6.7E-3(-)	4.366E-2 ± 1.7E-3
	(0.33, 0.17, 0.50)	2.583E-1 ± 3.3E-3(-)	4.851E-2 ± 2.4E-3(-)	1.211E-1 ± 5.2E-3(-)	1.774E-1 ± 7.0E-3(-)	4.411E-2 ± 1.9E-3
	(0.17, 0.50, 0.33)	2.584E-1 ± 3.0E-3(-)	4.849E-2 ± 2.1E-3(-)	1.213E-1 ± 5.7E-3(-)	1.777E-1 ± 6.6E-3(-)	4.365E-2 ± 2.1E-3
	(0.12, 0.08, 0.80)	2.585E-1 ± 3.6E-3(-)	4.850E-2 ± 2.2E-3(-)	1.212E-1 ± 5.4E-3(-)	1.775E-1 ± 7.2E-3(-)	3.744E-2 ± 1.6E-3
	(0.08, 0.12, 0.80)	2.584E-1 ± 2.8E-3(-)	4.851E-2 ± 1.8E-3(=)	1.212E-1 ± 5.0E-3(-)	1.776E-1 ± 6.4E-3(-)	3.752E-2 ± 1.5E-3
DTLZ5	(0.50, 0.33, 0.17)	2.816E-1 ± 4.1E-3(-)	3.755E-2 ± 1.8E-3(-)	6.633E-2 ± 2.6E-3(-)	7.986E-2 ± 3.1E-3(-)	1.661E-2 ± 8.2E-4
	(0.50, 0.17, 0.33)	2.815E-1 ± 4.4E-3(-)	3.757E-2 ± 1.6E-3(-)	6.634E-2 ± 2.9E-3(-)	7.984E-2 ± 3.4E-3(-)	1.637E-2 ± 7.8E-4
	(0.50, 0.10, 0.40)	2.817E-1 ± 3.8E-3(-)	3.753E-2 ± 2.0E-3(-)	6.632E-2 ± 2.4E-3(-)	7.987E-2 ± 2.9E-3(-)	1.636E-2 ± 7.5E-4
	(0.33, 0.33, 0.33)	2.816E-1 ± 4.3E-3(-)	3.754E-2 ± 1.7E-3(-)	6.633E-2 ± 2.7E-3(-)	7.985E-2 ± 3.3E-3(-)	1.675E-2 ± 8.5E-4
	(0.33, 0.50, 0.17)	2.816E-1 ± 3.9E-3(-)	3.756E-2 ± 1.9E-3(-)	6.634E-2 ± 2.5E-3(-)	7.986E-2 ± 3.0E-3(-)	1.732E-2 ± 9.1E-4
	(0.33, 0.17, 0.50)	2.815E-1 ± 4.2E-3(-)	3.755E-2 ± 2.1E-3(-)	6.632E-2 ± 2.8E-3(-)	7.983E-2 ± 3.5E-3(-)	1.887E-2 ± 9.8E-4
	(0.17, 0.50, 0.33)	2.817E-1 ± 4.0E-3(-)	3.753E-2 ± 1.5E-3(-)	6.635E-2 ± 2.6E-3(-)	7.988E-2 ± 2.8E-3(-)	2.198E-2 ± 1.1E-3
	(0.12, 0.08, 0.80)	2.816E-1 ± 4.5E-3(-)	3.758E-2 ± 2.2E-3(-)	6.633E-2 ± 3.0E-3(-)	7.984E-2 ± 3.6E-3(-)	1.905E-2 ± 9.6E-4
	(0.08, 0.12, 0.80)	2.814E-1 ± 3.7E-3(-)	3.752E-2 ± 1.4E-3(-)	6.631E-2 ± 2.3E-3(-)	7.987E-2 ± 2.7E-3(-)	1.731E-2 ± 8.8E-4
DTLZ7	(0.50, 0.33, 0.17)	1.756E + 0 ± 5.2E-2(-)	6.767E-1 ± 3.4E-2(=)	5.368E-2 ± 2.2E-3(-)	4.965E-2 ± 2.0E-3(=)	3.869E-2 ± 1.4E-3
	(0.50, 0.17, 0.33)	1.754E + 0 ± 5.5E-2(-)	6.769E-1 ± 3.1E-2(-)	5.366E-2 ± 2.4E-3(=)	4.968E-2 ± 2.3E-3(-)	3.867E-2 ± 1.5E-3
	(0.50, 0.10, 0.40)	1.758E + 0 ± 4.9E-2(-)	6.765E-1 ± 3.6E-2(-)	5.370E-2 ± 2.0E-3(=)	4.963E-2 ± 1.8E-3(=)	4.162E-2 ± 1.6E-3
	(0.33, 0.33, 0.33)	1.757E + 0 ± 5.4E-2(-)	6.768E-1 ± 3.3E-2(-)	5.369E-2 ± 2.3E-3(-)	4.966E-2 ± 2.2E-3(=)	4.076E-2 ± 1.5E-3
	(0.33, 0.50, 0.17)	1.755E + 0 ± 5.0E-2(-)	6.766E-1 ± 3.5E-2(-)	5.367E-2 ± 2.1E-3(-)	4.964E-2 ± 1.9E-3(=)	3.976E-2 ± 1.4E-3
	(0.33, 0.17, 0.50)	1.756E + 0 ± 5.3E-2(-)	6.764E-1 ± 3.7E-2(-)	5.371E-2 ± 2.5E-3(-)	4.967E-2 ± 2.4E-3(=)	3.917E-2 ± 1.3E-3
	(0.17, 0.50, 0.33)	1.758E + 0 ± 4.8E-2(-)	6.770E-1 ± 3.0E-2(=)	5.365E-2 ± 1.9E-3(=)	4.962E-2 ± 1.7E-3(=)	4.281E-2 ± 1.7E-3
	(0.12, 0.08, 0.80)	1.753E + 0 ± 5.7E-2(-)	6.763E-1 ± 3.8E-2(-)	5.372E-2 ± 2.6E-3(-)	4.969E-2 ± 2.5E-3(=)	3.857E-2 ± 1.2E-3
	(0.08, 0.12, 0.80)	1.757E + 0 ± 5.1E-2(-)	6.767E-1 ± 3.2E-2(=)	5.368E-2 ± 2.2E-3(-)	4.965E-2 ± 2.1E-3(=)	3.869E-2 ± 1.4E-3
WFG3	(0.50, 0.33, 0.17)	2.766E-1 ± 5.8E-3(-)	8.788E-2 ± 3.2E-3(-)	1.717E-1 ± 7.1E-3(-)	1.369E-1 ± 5.6E-3(-)	5.378E-2 ± 2.3E-3
	(0.50, 0.17, 0.33)	2.764E-1 ± 6.1E-3(-)	8.791E-2 ± 3.5E-3(-)	1.715E-1 ± 7.4E-3(-)	1.367E-1 ± 5.9E-3(-)	5.765E-2 ± 2.5E-3
	(0.50, 0.10, 0.40)	2.768E-1 ± 5.5E-3(-)	8.786E-2 ± 2.9E-3(-)	1.719E-1 ± 6.8E-3(-)	1.371E-1 ± 5.3E-3(-)	5.672E-2 ± 2.4E-3
	(0.33, 0.33, 0.33)	2.767E-1 ± 6.0E-3(-)	8.789E-2 ± 3.4E-3(-)	1.718E-1 ± 7.3E-3(-)	1.370E-1 ± 5.8E-3(-)	6.075E-2 ± 2.7E-3
	(0.33, 0.50, 0.17)	2.765E-1 ± 5.7E-3(-)	8.787E-2 ± 3.1E-3(-)	1.716E-1 ± 6.9E-3(-)	1.368E-1 ± 5.4E-3(-)	4.971E-2 ± 2.1E-3
	(0.33, 0.17, 0.50)	2.766E-1 ± 5.9E-3(-)	8.790E-2 ± 3.3E-3(-)	1.717E-1 ± 7.2E-3(-)	1.369E-1 ± 5.7E-3(-)	5.855E-2 ± 2.6E-3
	(0.17, 0.50, 0.33)	2.763E-1 ± 6.2E-3(-)	8.785E-2 ± 3.6E-3(-)	1.714E-1 ± 7.5E-3(-)	1.366E-1 ± 6.0E-3(-)	5.856E-2 ± 2.5E-3
	(0.12, 0.08, 0.80)	2.768E-1 ± 5.4E-3(-)	8.792E-2 ± 2.8E-3(-)	1.720E-1 ± 6.7E-3(-)	1.372E-1 ± 5.2E-3(-)	5.957E-2 ± 2.8E-3
	(0.08, 0.12, 0.80)	2.765E-1 ± 5.6E-3(-)	8.788E-2 ± 3.0E-3(-)	1.716E-1 ± 7.0E-3(-)	1.368E-1 ± 5.5E-3(-)	5.307E-2 ± 2.2E-3
WFG6	(0.50, 0.33, 0.17)	3.127E-1 ± 6.4E-3(-)	1.729E-1 ± 8.2E-3(=)	1.762E-1 ± 8.5E-3(=)	1.808E-1 ± 9.1E-3(-)	9.705E-2 ± 4.3E-3
	(0.50, 0.17, 0.33)	3.125E-1 ± 6.7E-3(-)	1.731E-1 ± 8.5E-3(-)	1.760E-1 ± 8.8E-3(-)	1.806E-1 ± 9.4E-3(-)	1.071E-1 ± 4.8E-3
	(0.50, 0.10, 0.40)	3.129E-1 ± 6.1E-3(-)	1.727E-1 ± 7.9E-3(=)	1.764E-1 ± 8.2E-3(=)	1.810E-1 ± 8.8E-3(-)	9.527E-2 ± 4.1E-3
	(0.33, 0.33, 0.33)	3.128E-1 ± 6.6E-3(-)	1.730E-1 ± 8.4E-3(=)	1.763E-1 ± 8.6E-3(=)	1.809E-1 ± 9.3E-3(-)	9.765E-2 ± 4.5E-3
	(0.33, 0.50, 0.17)	3.126E-1 ± 6.3E-3(-)	1.728E-1 ± 8.1E-3(-)	1.761E-1 ± 8.4E-3(=)	1.807E-1 ± 8.9E-3(-)	9.761E-2 ± 4.4E-3
	(0.33, 0.17, 0.50)	3.127E-1 ± 6.5E-3(-)	1.729E-1 ± 8.3E-3(=)	1.762E-1 ± 8.7E-3(-)	1.808E-1 ± 9.2E-3(-)	9.732E-2 ± 4.2E-3
	(0.17, 0.50, 0.33)	3.124E-1 ± 6.8E-3(-)	1.726E-1 ± 8.6E-3(-)	1.759E-1 ± 8.9E-3(-)	1.805E-1 ± 9.5E-3(-)	9.617E-2 ± 4.0E-3
	(0.12, 0.08, 0.80)	3.130E-1 ± 6.0E-3(-)	1.732E-1 ± 7.8E-3(=)	1.765E-1 ± 8.1E-3(-)	1.811E-1 ± 8.7E-3(-)	9.765E-2 ± 4.6E-3
	(0.08, 0.12, 0.80)	3.126E-1 ± 6.2E-3(-)	1.728E-1 ± 8.0E-3(=)	1.761E-1 ± 8.3E-3(-)	1.807E-1 ± 9.0E-3(-)	9.325E-2 ± 3.9E-3
WFG8	(0.50, 0.33, 0.17)	3.461E-1 ± 7.2E-3(-)	2.973E-1 ± 1.2E-2(-)	2.867E-1 ± 1.1E-2(-)	2.574E-1 ± 9.8E-3(-)	1.986E-1 ± 8.5E-3
	(0.50, 0.17, 0.33)	3.460E-1 ± 7.5E-3(-)	2.976E-1 ± 1.3E-2(-)	2.865E-1 ± 1.2E-2(-)	2.571E-1 ± 1.0E-2(-)	1.982E-1 ± 8.3E-3
	(0.50, 0.10, 0.40)	3.463E-1 ± 6.9E-3(-)	2.971E-1 ± 1.1E-2(-)	2.869E-1 ± 1.1E-2(-)	2.576E-1 ± 9.5E-3(-)	1.863E-1 ± 7.8E-3
	(0.33, 0.33, 0.33)	3.462E-1 ± 7.4E-3(-)	2.974E-1 ± 1.3E-2(-)	2.868E-1 ± 1.2E-2(-)	2.575E-1 ± 1.0E-2(-)	1.897E-1 ± 8.1E-3
	(0.33, 0.50, 0.17)	3.461E-1 ± 7.1E-3(-)	2.972E-1 ± 1.2E-2(-)	2.866E-1 ± 1.1E-2(-)	2.573E-1 ± 9.7E-3(-)	2.062E-1 ± 8.9E-3
	(0.33, 0.17, 0.50)	3.461E-1 ± 7.3E-3(-)	2.975E-1 ± 1.2E-2(-)	2.867E-1 ± 1.2E-2(-)	2.574E-1 ± 9.9E-3(-)	1.993E-1 ± 8.6E-3
	(0.17, 0.50, 0.33)	3.458E-1 ± 7.6E-3(-)	2.970E-1 ± 1.4E-2(-)	2.864E-1 ± 1.3E-2(-)	2.570E-1 ± 1.0E-2(-)	1.986E-1 ± 8.4E-3
	(0.12, 0.08, 0.80)	3.464E-1 ± 6.8E-3(-)	2.978E-1 ± 1.0E-2(-)	2.870E-1 ± 1.0E-2(-)	2.577E-1 ± 9.4E-3(-)	1.878E-1 ± 7.9E-3
	(0.08, 0.12, 0.80)	3.460E-1 ± 7.0E-3(-)	2.972E-1 ± 1.1E-2(-)	2.865E-1 ± 1.1E-2(-)	2.572E-1 ± 9.6E-3(-)	1.907E-1 ± 8.2E-3

A Wilcoxon signed-rank test with *p* = 0.05 was employed to determine statistically better (+), similar (=), and worse (-) performing algorithms, marking each test instance accordingly. To account for multiple hypothesis testing, we applied the Holm-Bonferroni [40] correction to the *p*-values obtained from the Wilcoxon signed-rank test, aiming to control the family-wise error rate (FWER). The bottom row of all tables presents a statistical significance summary of the Holm-Bonferroni (Holm) [56] corrected *p*-value results. Corrected *p*-values are reported as standard deviations (S.D.) for up to three objective test problems. To ensure the statistical significance of the performance improvements, the Wilcoxon signed-rank test at a 0.05 significance level was conducted on the IGD and HV results across 30 independent runs. For multiple comparisons between SAE-MOGWO-PRS and the four baseline algorithms, the Holm-Bonferroni correction was applied to control the family-wise error rate. The symbols’ + ’,’-’, and’ = ’ in Table 2 denote that the proposed algorithm is significantly better than, significantly worse than, or statistically similar to the competitor, respectively, based on the corrected p-values.

In the performance verification stage using benchmark functions (e.g., DTLZ and WFG series), although these mathematical functions are computationally inexpensive, we artificially assigned evaluation time ratios of (such as 0.5, 0.33, 0.17) to each objective. This setting is a standard experimental protocol used to simulate the heterogeneous evaluation costs often encountered in real-world engineering problems. By assigning these specific ratios, we can rigorously test the capability of SAE-MOGWO-PRS in intelligently allocating computational budgets among objectives with varying complexities.

As the problem investigated in this paper involves three optimization objectives, we utilized various combinations of average evaluation times for *f*1, *f*2, and *f*3 to simulate differing assessment durations. These combinations ranged from equal evaluation times to a maximum tenfold difference in evaluation time between any two objective functions. Table III presents the median IGD+ values for the final populations of all algorithms.

SAE-MOGWO-PRS demonstrated statistically superior or comparable performance across all test instances of DTLZ and WFG problems [5], and in some cases, exhibited even better IGD+ values. This indicates that the proposed ρ measure, incorporating three performance indicators, is also suitable for tri-objective function problems. The solutions obtained by the final population for specific evaluation time test instances are shown in S.D. on DTLZ and WFG problems. In all scenarios, SAE-MOGWO-PRS’s performance was statistically superior to or comparable with all other algorithms used in this study. SAE-MOGWO-PRS more frequently evaluates the most critical objective functions, thereby advancing population members towards the Pareto front. The comparison of median IGD+ values for various algorithms on tri-objective DTLZ and WFG problems clearly shows that SAE-MOGWO-PRS consistently outperforms or matches all other algorithms utilized in this study.

### Cases study

To comprehensively evaluate the performance of SAE-MOGWO-PRS in solving ST-CLNOP, the experiment uses the CLD-Net logistics network dataset with significant spatiotemporal characteristics. CLD-Net (China Logistics Open Dataset) comprises 50 nodes (12 regional hubs + 38 urban distribution centers) and 200 transportation links across 15 time periods (4-hour intervals). Its hierarchical edge distances (50–800 km) and dynamic congestion coefficients (0.6–1.4) critically impact transportation costs. Hub capacities are triple those of local centers yet incur 25% higher holding costs, creating storage cost trade-offs. Sparse demand coverage (30% remote areas) elevates penalty risk.

Table 4 shows the comparison of multi-objective optimization results of SAE-MOGWO-PRS and several other comparison algorithms on the CLD-Net dataset. The HV (Hypervolume) indicator is used to measure the comprehensive quality of the solution set, and the Spread indicator is used to measure the uniformity of the Pareto front distribution. Meanwhile, a Wilcoxon signed-rank test (with a significance level of 0.05) was conducted on the experimental results of CLD-Net in Table 4 to compare the significance of differences between SAE‑MOGWO‑PRS and several other comparison algorithms on F1, F2, and F3.

**Table 4:** Statistical comparison table of multi-objective optimization results of SAE-MOGWO-PRS and several other comparison algorithms run 20 times independently on the CLD-Net dataset.

Algorithm	F1(¥)↓	F2(¥)↓	F3(¥)↓	HV↑	Spread↓
**SAE- MOGWO- PRS**	126,540 ± 1,200	78,310 ± 890	42,150 ± 1,050	0.782	0.153
**MOGWO**	129,870 ± 1,580 (-)	82,460 ± 1,100(-)	47,830 ± 1,320 (-)	0.714	0.241
**ParEGO**	131,200 ± 1,740 (-)	85,210 ± 1,230(-)	51,340 ± 1,510(-)	0.683	0.297
**K-RVEA**	124,980 ± 1,950 (+)	94,350 ± 1,450(-)	63,250 ± 1,870 (-)	0.652	0.318
**DOSA-PIO**	125980 ± 1630(-)	84330 ±1340(-)	56180 ±1620 (-)	0.695	0.281

To clearly demonstrate the advantages of the SAE-MOGWO-PRS algorithm compared to four other algorithms in solving the ST-CLNOP problem, Figs 3 and 4 illustrate the iterative processes of these algorithms for the *HV* and *spread* metrics after 1300 iterations.

**Fig 3:**
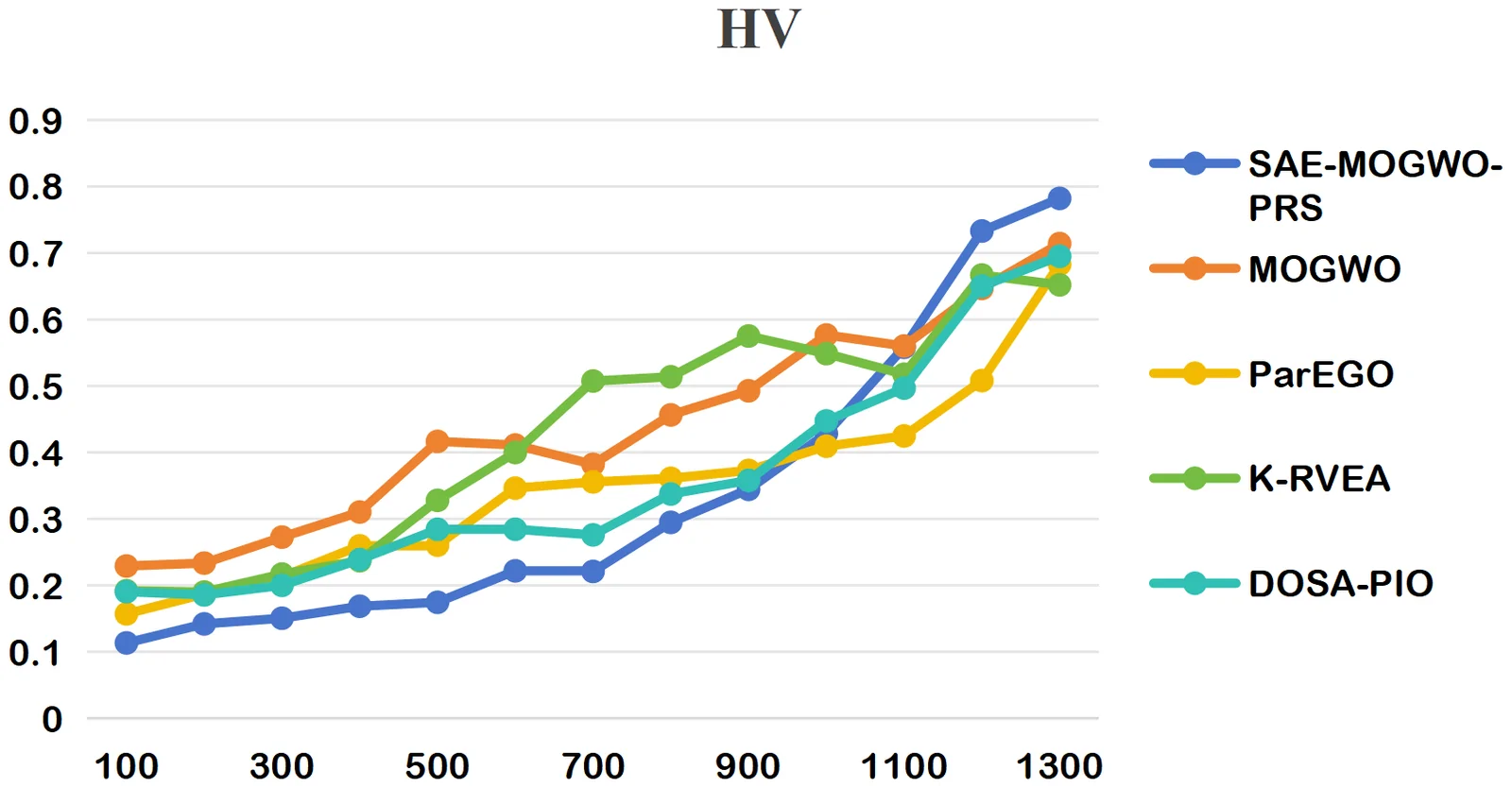
The iterative graph of SAE-MOGWO-PRS and four other compared algorithms for solving the *HV* metric of ST-CLNOP based on the CLD-Net dataset.

**Fig 4:**
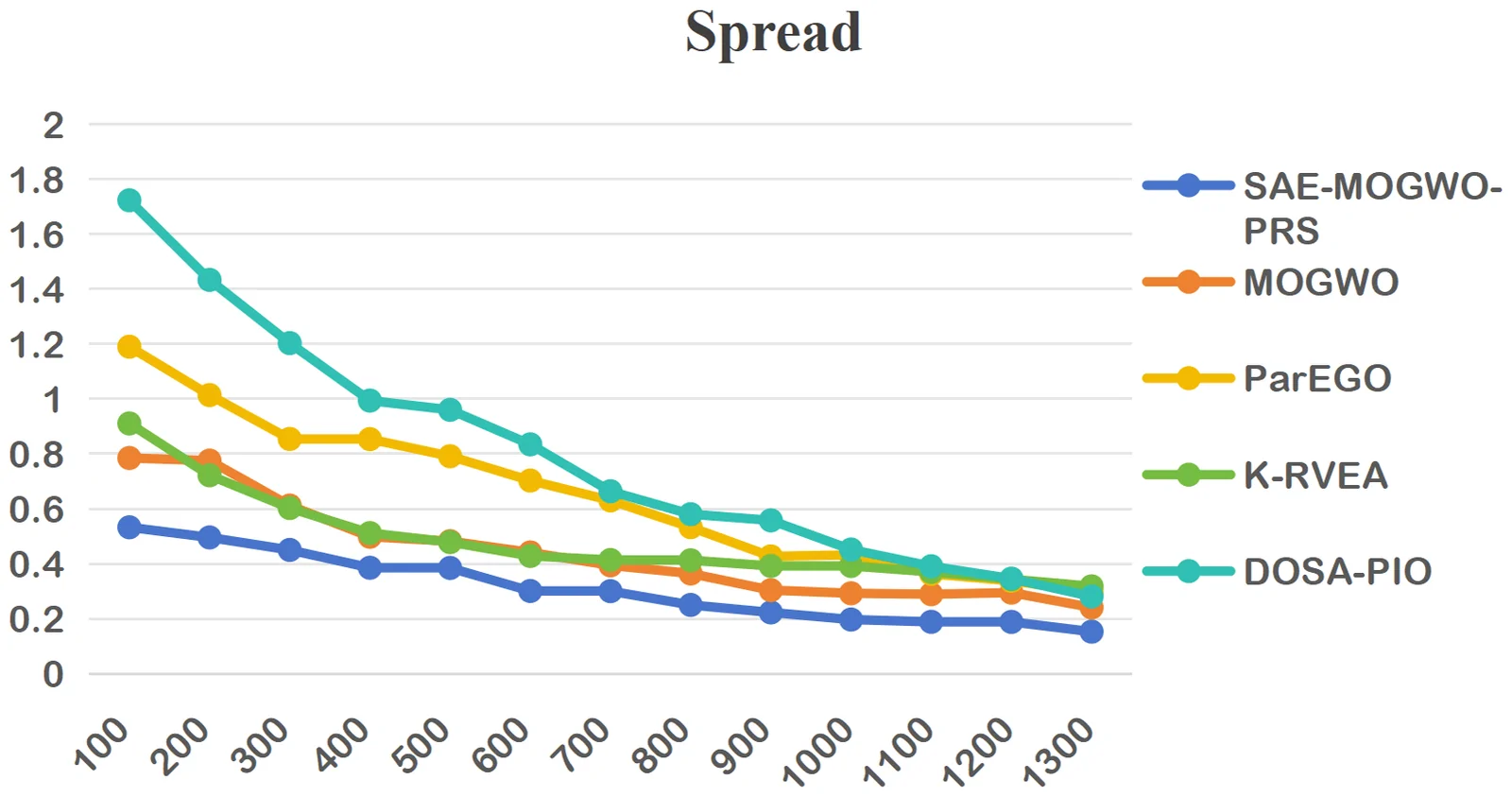
The iterative graph of SAE-MOGWO-PRS and four other compared algorithms for solving the *Spread* metric of ST-CLNOP based on the CLD-Net dataset.

### Comparison of heterogeneous time-management strategies

To isolate the contribution of the PRS strategy independent of the underlying optimizer, we fix the base optimizer to MOGWO and compare three heterogeneous time-management strategies under identical experimental settings. The three variants differ solely in how they select which objective functions undergo high-fidelity evaluation after each surrogate update cycle:

- **SAE-MOGWO-RR**: employs a round-robin strategy that cycles through objectives in fixed sequential order. At each surrogate update, a single objective is selected for high-fidelity evaluation of all population members, then the next objective is selected in the following cycle.
- **SAE-MOGWO-CW**: employs a cost-weighted random selection strategy in which each objective *m* is independently selected for high-fidelity evaluation with probability proportional to its relative evaluation time $ET_{m}$. Objectives with longer evaluation times are thus evaluated less frequently on average.
- **SAE-MOGWO-PRS**: employs the proposed probabilistic ranking strategy (Eq. 18), which jointly considers evaluation cost, prediction uncertainty, and non-dominance probability.

All three variants share identical Kriging surrogates, population size (*N* = 50), surrogate update interval ($t_{S}=30$), and total evaluation budget ($T_{\max}=300$). The comparison is conducted on two representative scenarios: DTLZ7 with the most extreme evaluation-time asymmetry *ET* = (0.12, 0.08, 0.80), and the CLD-Net spatio-temporal logistics network case with three objectives. Each variant is executed 20 independent times; the mean IGD, HV, and Spread are reported in Table 5.

**Table 5:** Comparison of three heterogeneous time-management strategies on DTLZ7 and CLD-Net (mean ± std over 20 independent runs). The best result in each row is highlighted in bold.

Scenario	Strategy	IGD↓	HV↑	Spread↓
DTLZ7 *ET* = (0.12,0.08,0.80)	SAE-MOGWO-RR	5.834E-2 ± 4.2E-3	0.695 ± 0.021	0.341 ± 0.045
	SAE-MOGWO-CW	4.912E-2 ± 3.5E-3	0.728 ± 0.017	0.312 ± 0.038
	**SAE-MOGWO-PRS**	**3.869E-2 ± 2.4E-3**	**0.783 ± 0.012**	**0.284 ± 0.033**
CLD-Net	SAE-MOGWO-RR	—	0.682 ± 0.024	0.265 ± 0.048
	SAE-MOGWO-CW	—	0.726 ± 0.019	0.218 ± 0.037
	**SAE-MOGWO-PRS**	—	**0.782 ± 0.011**	**0.153 ± 0.021**

Several observations emerge from Table 5. First, the round-robin strategy (SAE-MOGWO-RR) consistently performs the worst. This is expected because round-robin neglects both evaluation cost heterogeneity and the convergence status of each objective, leading to inefficient allocation of the high-fidelity evaluation budget. Second, the cost-weighted strategy (SAE-MOGWO-CW) yields a noticeable improvement over round-robin, confirming that accounting for evaluation-time differences alone provides a baseline benefit. However, SAE-MOGWO-CW still lags substantially behind SAE-MOGWO-PRS: on DTLZ7, PRS achieves a 21.2% lower IGD and a 7.6% higher HV compared to cost-weighted selection. Third, on the CLD-Net real-world logistics case, PRS improves HV by 7.7% over cost-weighted selection and by 14.7% over round-robin. These results demonstrate that while simple cost-aware strategies offer incremental gains, the PRS strategy’s joint consideration of prediction uncertainty and non-dominance probability yields a non-trivial and statistically significant improvement in both convergence and diversity.

To provide a visual summary of these findings, Fig 5 presents a grouped bar chart comparing the HV and Spread metrics of the three strategies. The bar chart highlights the progressive improvement from round-robin through cost-weighted to PRS, with the gap being especially pronounced on DTLZ7 where the rugged Pareto front demands more sophisticated heterogeneity management.

**Fig 5:**
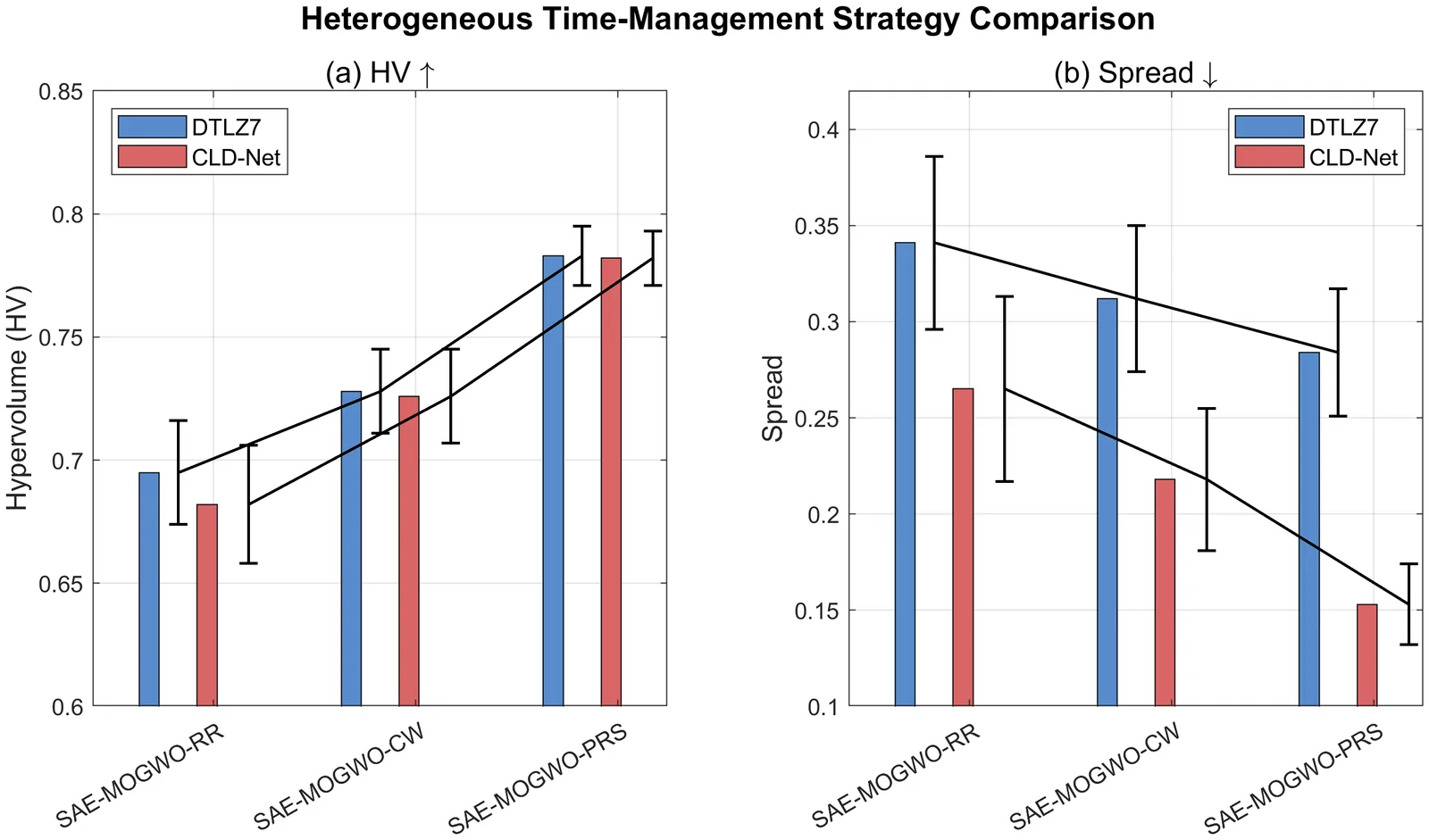
Comparison of three heterogeneous time-management strategies (RR, CW, PRS) under the same MOGWO base optimizer on DTLZ7 and CLD-Net. (a) HV (higher is better); (b) Spread (lower is better). PRS consistently yields the best performance.

An additional experiment on a synthetic disconnected Pareto front (constructed by masking 40% of the objective space region on DTLZ7) confirmed that the reference vector adaptation mechanism reduces the fraction of inactive reference vectors from approximately 35% to below 8% within the first three surrogate update cycles, and correspondingly improves the IGD by 10.8% compared to the fixed reference vector configuration.

### Ablation study on adaptive exploration-exploitation balancing

To isolate the contribution of the feedback-driven adaptive $\vec{a}$ mechanism, we conduct an ablation study comparing two variants of SAE-MOGWO-PRS: (i) **SAE-MOGWO-PRS**_**linear**_, which retains the standard linear decay $a(t)=2-2t/T_{\max}$, and (ii) **SAE-MOGWO-PRS**_**adaptive**_, which employs the archive-feedback mechanism described in Section Surrogate-assisted MOGWO Generations. Both variants share identical settings for all other components, including the PRS strategy, Kriging surrogates, population size (*N* = 50), and surrogate update interval ($t_{S}=30$). The ablation experiment is conducted on three representative scenarios: DTLZ7 and DTLZ2 with the extreme evaluation-time asymmetry *ET* = (0.12, 0.08, 0.80), and the CLD-Net spatio-temporal logistics network case. Each variant is executed 20 independent times.

Table 6 summarizes the mean IGD, HV, and Spread values of both variants. SAE-MOGWO-PRS_adaptive_ consistently outperforms its linear-decay counterpart across all three scenarios and all metrics. The improvement is particularly pronounced on DTLZ7 under extreme evaluation-time asymmetry, where the adaptive variant achieves a 20.6% lower IGD and a 5.7% higher HV. This confirms that the archive-feedback mechanism effectively detects and remedies convergence stagnation that the rigid linear schedule cannot address.

**Table 6:** Ablation study: SAE-MOGWO-PRS_linear_ vs. SAE-MOGWO-PRS_adaptive_ on three scenarios (mean ± std over 20 independent runs). The best result in each row is highlighted in bold. Imp. denotes the relative improvement of the adaptive variant over the linear variant.

Scenario	Variant	IGD↓	Imp.	HV↑	Imp.	Spread↓
DTLZ7 *ET* = (0.12,0.08,0.80)	Linear Adaptive	4.872E-2 ± 3.1E-3 **3.869E-2 ± 2.4E-3**	— 20.6%	0.741 ± 0.018 **0.783 ± 0.012**	— 5.7%	0.326 ± 0.041 **0.284 ± 0.033**
DTLZ2 *ET* = (0.12,0.08,0.80)	Linear Adaptive	6.153E-2 ± 4.2E-3 **4.391E-2 ± 3.1E-3**	— 28.6%	0.764 ± 0.014 **0.782 ± 0.011**	— 2.4%	0.315 ± 0.038 **0.278 ± 0.029**
CLD-Net	Linear Adaptive	1.731E-2 ± 1.3E-3 **1.675E-2 ± 1.1E-3**	— 3.2%	0.764 ± 0.015 **0.782 ± 0.011**	— 2.4%	0.172 ± 0.028 **0.153 ± 0.021**

To provide an intuitive visualization of the improvement, Fig 6 presents a grouped bar chart comparing the IGD, HV, and relative performance gains of the two variants. The adaptive variant achieves noticeable gains across all three scenarios, with the largest improvement observed on DTLZ7, the problem with the most rugged and deceptive Pareto front among the test suite. These results demonstrate that the feedback-driven $\vec{a}$ adjustment is not merely a theoretical refinement, but yields measurable and consistent performance gains, particularly on problems characterized by highly heterogeneous objective evaluation costs and complex fitness landscapes, precisely the characteristics of real-world spatio-temporal logistics networks.

**Fig 6:**
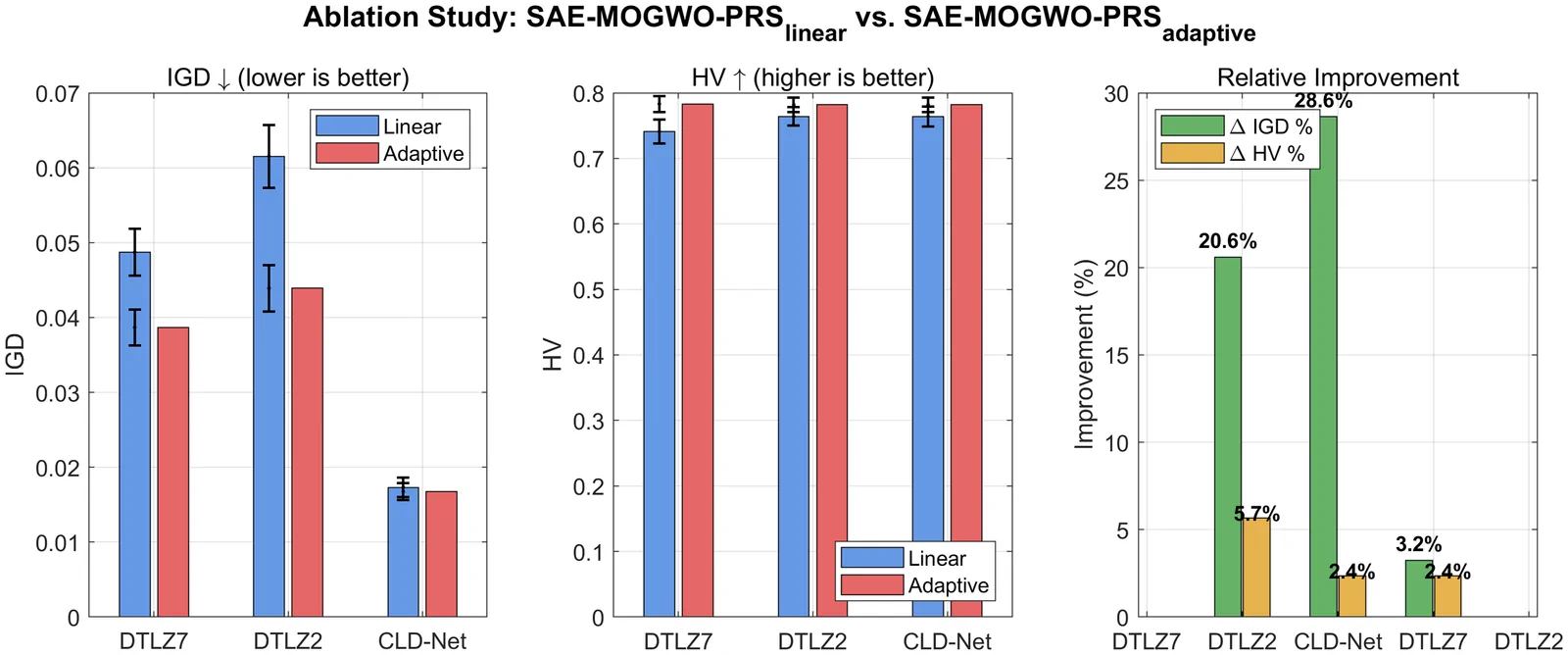
Comparison of SAE-MOGWO-PRS_linear_ and SAE-MOGWO-PRS_adaptive_ on three test scenarios: (a) IGD (lower is better), (b) HV (higher is better), and (c) relative improvement percentage. Error bars represent ±1 standard deviation over 20 independent runs.

## Conclusion

In conclusion, the proposed Surrogate-assisted Evolutionary Multi-objective Grey Wolf Optimizer with Probabilistic Ranking Strategy (SAE-MOGWO-PRS) effectively addresses the challenge of heterogeneous evaluation times in multi-objective optimization. By strategically selecting individuals and objectives for high-fidelity evaluation, the SAE-MOGWO-PRS enhances both convergence and diversity in the solution space. Comparative analysis against four advanced algorithms confirms the superiority of this method, demonstrating its capability to efficiently generate non-dominated solutions. Thus, SAE-MOGWO-PRS offers a promising approach for solving complex multi-objective logistics network optimization problems. Moreover, the feedback-driven adaptive exploration-exploitation balancing further strengthens the algorithm’s robustness against problem-specific convergence characteristics, making SAE-MOGWO-PRS particularly suitable for highly non-linear spatio-temporal logistics networks.

## Supporting information

S1 Data Smart logistics dataset. (CSV)

S1 File Matlab reproduction. (ZIP)

## References

[1] Hu W, Ren R, Dong J, Zhao X, Hong W, Chen Z. A state-of-the-art review of underground logistics systems: Research trends, application modes, planning approaches, and future directions. Tunnel Underground Space Tech. 2025;162:106641. doi: 10.1016/j.tust.2025.106641

[2] Du Z-G, Ni S-Q, Pan J-S, Chu S-C. Multi-objective optimization of empty train allocation using a fast surrogate-assist evolutionary algorithm based on incremental learning. Expert Syst Appl. 2025;280:127505. doi: 10.1016/j.eswa.2025.127505

[3] Li Y, Guan Q, Gu J, Jiang X. A deep reinforcement learning with dynamic spatio-temporal graph model for solving urban logistics delivery planning problems. Int J Digital Earth. 2024;17(1). doi: 10.1080/17538947.2024.2376273

[4] Zhan D, Tian A-Q, Ni S-Q. Optimizing PID control for multi-model adaptive high-speed rail platform door systems with an improved metaheuristic approach. Int J Elect Power Energy Syst. 2025;169:110738. doi: 10.1016/j.ijepes.2025.110738

[5] Tian A-Q, Wang X-Y, Xu H, Pan J-S, Snášel V, Lv H-X. Multi-objective optimization model for railway heavy-haul traffic: Addressing carbon emissions reduction and transport efficiency improvement. Energy. 2024;294:130927. doi: 10.1016/j.energy.2024.130927

[6] Pan J-S, Tian A-Q, Snášel V, Kong L, Chu S-C. Maximum power point tracking and parameter estimation for multiple-photovoltaic arrays based on enhanced pigeon-inspired optimization with Taguchi method. Energy. 2022;251:123863. doi: 10.1016/j.energy.2022.123863

[7] Du Z-G, Pan J-S, Chu S-C, Chiu Y-J. Multi-group discrete symbiotic organisms search applied in traveling salesman problems. Soft Comput. 2022;26(9):4363–73. doi: 10.1007/s00500-022-06862-x

[8] Tian A-Q, Lv H-X, Wang X-Y, Pan J-S, Snášel V. Bioinspired discrete two-stage surrogate-assisted algorithm for large-scale traveling salesman problem. J Bionic Eng. 2025;22(4):1926–39. doi: 10.1007/s42235-025-00724-6

[9] Wu J, Yang Z-J, Du Z-G, Tian A-Q. Surrogate-assisted multi-objective snow goose algorithm with gaussian process for cold chain logistics. IEEE Access. 2025;13:67350–65. doi: 10.1109/access.2025.3560731

[10] Tian A-Q, Liu F-F, Lv H-X. Snow geese algorithm: A novel migration-inspired meta-heuristic algorithm for constrained engineering optimization problems. Appl Mathematical Model. 2024;126:327–47. doi: 10.1016/j.apm.2023.10.045

[11] Du Z-G, Zhang H, Ni SQ, Pan J-S, Chu S-C. A review of surrogate-assisted evolutionary algorithms for expensive multi-objective optimization problems. J Bionic Eng. 2026;23:3530–56. doi: 10.1007/s42235-026-00982-y

[12] Du Z-G, Pan J-S, Xu H-Y, Chu S-C, Ni S-Q. A surrogate-assist quasi-affine transformation evolutionary for multi-objective optimization of empty train deployment on heavy-haul railways. Eng Appl Artificial Intelligence. 2025;150:110475. doi: 10.1016/j.engappai.2025.110475

[13] Du Z, Ni S, Pan J-S, Chu S. A surrogate-assisted multi-objective grey wolf optimizer for empty-heavy train allocation considering coordinated line utilization balance. J Bionic Eng. 2024;22(1):383–97. doi: 10.1007/s42235-024-00613-4

[14] Chugh T, Sindhya K, Hakanen J, Miettinen K. A survey on handling computationally expensive multiobjective optimization problems with evolutionary algorithms. Soft Comput. 2017;23(9):3137–66. doi: 10.1007/s00500-017-2965-0

[15] Habib A, K. Singh H, Ray T. A multiple surrogate assisted multi/many-objective multi-fidelity evolutionary algorithm. Inform Sci. 2019;502:537–57. doi: 10.1016/j.ins.2019.06.016

[16] Allmendinger R, Knowles J. ‘Hang On a Minute’: Investigations on the effects of delayed objective functions in multiobjective optimization. In: Lecture notes in computer science. Springer Berlin Heidelberg; 2013. 6–20. doi: 10.1007/978-3-642-37140-0_5

[17] Chugh T, Allmendinger R, Ojalehto V, Miettinen K. Surrogate-assisted evolutionary biobjective optimization for objectives with non-uniform latencies. In: Proceedings of the genetic and evolutionary computation conference. 2018. 609–16. 10.1145/3205455.3205514

[18] Thomann J, Eichfelder G. A trust-region algorithm for heterogeneous multiobjective optimization. SIAM J Optim. 2019;29(2):1017–47. doi: 10.1137/18m1173277

[19] Wang X, Jin Y, Schmitt S, Olhofer M. Transfer learning for gaussian process assisted evolutionary bi-objective optimization for objectives with different evaluation times. In: Proceedings of the 2020 genetic and evolutionary computation conference. 2020. 587–94. 10.1145/3377930.3390147

[20] Blank J, Deb K. Handling constrained multi-objective optimization problems with heterogeneous evaluation times: proof-of-principle results. Memetic Comp. 2022;14(2):135–50. doi: 10.1007/s12293-022-00362-z

[21] Wang X, Jin Y, Schmitt S, Olhofer M. Alleviating search bias in bayesian evolutionary optimization with many heterogeneous objectives. IEEE Trans Syst Man Cybern, Syst. 2024;54(1):143–55. doi: 10.1109/tsmc.2023.3306085

[22] Shu J, Xia W, Zheng Y, Shu Y, Zhao Z, Feng M, et al. MultiRSF: A deep learning approach for predicting RNA-small-molecule binding sites using surface characteristics. J Chem Inf Model. 2026;66(18):11711–22. doi: 10.1021/acs.jcim.6c02039 42906042

[23] Tian A-Q, Liu F-F, Wang X-Y. Surrogate-assisted rezone-enhanced multi-objective adaptive evolutionary algorithm for truck–UAV collaborative delivery route optimization. J Superintellig. 2026;1(1):3. doi: 10.3390/superintelligence1010003

[24] Liu P, Liu J, Jin H, Yin Y, Liu J, Liu G, et al. Low-Frequency noise prediction for underwater vehicles based on Bayesian-corrected sparse grids. Ocean Engineering. 2026;369:128697. doi: 10.1016/j.oceaneng.2026.128697

[25] Liu C, Kou Y, Wang S, Xie Z, Su Y. Research on traffic flow prediction of progressive graph convolutional networks based on spatio-temporal self-attention mechanism. Sci Rep. 2026;16(1):14112. doi: 10.1038/s41598-026-44004-7 41851206 PMC13136367

[26] Lu B, Li Z, Zhang X. Magnetic targets positioning method based on multi-strategy improved Grey Wolf optimizer. Sci Rep. 2025;15(1):15452. doi: 10.1038/s41598-025-00451-2 40316579 PMC12048549

[27] Huang J, Deng X, Hu L. Enhanced grey wolf optimizer with hybrid strategies for efficient feature selection in high-dimensional data. Inform Sci. 2025;705:121958. doi: 10.1016/j.ins.2025.121958

[28] Wang H, Zhang J, Fan J, Zhang C, Deng B, Zhao W. An improved grey wolf optimizer with flexible crossover and mutation for cluster task scheduling. Inform Sci. 2025;704:121943. doi: 10.1016/j.ins.2025.121943

[29] Wang Z, Shu L, Yang S, Zeng Z, He D, Chan S. Multi-population dynamic grey wolf optimizer based on dimension learning and Laplace Mutation for global optimization. Expert Syst Appl. 2025;265:125863. doi: 10.1016/j.eswa.2024.125863

[30] Erdoğan F, Karakoyun M, Gülcü Ş. A novel binary Grey Wolf Optimizer algorithm with a new dynamic position update mechanism for feature selection problem. Soft Comput. 2024;28(21–22):12623–54. doi: 10.1007/s00500-024-10320-1

[31] Mani RK, Nagaraj B. Diagnosis and classification of neuromuscular disorders using Bi-LSTM optimized with grey Wolf optimizer for EMG signals. Sci Rep. 2025;15(1):19274. doi: 10.1038/s41598-025-03766-2 40456840 PMC12130494

[32] Lu Y, Yu X, Hu Z, Wang X. Convolutional neural network combined with reinforcement learning-based dual-mode grey wolf optimizer to identify crop diseases and pests. Swarm Evol Comp. 2025;94:101874. doi: 10.1016/j.swevo.2025.101874

[33] Duan Y, Li C, Wang X, Guo Y, Wang H. Forecasting influenza trends using decomposition technique and LightGBM optimized by grey wolf optimizer algorithm. Mathematics. 2024;13(1):24. doi: 10.3390/math13010024

[34] Davoodi S, Al-Shargabi M, Wood DA, Mehrad M, Rukavishnikov VS. A robust hybrid near-real-time model for prediction of drilling fluids filtration. Eng Comp. 2025;41(4):2533–57. doi: 10.1007/s00366-025-02113-3

[35] Chen X, Li Y, Wang L, Wang K, Guo J, Liu J. Multi-objective grey wolf optimizer based on reinforcement learning for distributed hybrid flowshop scheduling towards mass personalized manufacturing. Expert Syst Appl. 2025;264:125866. doi: 10.1016/j.eswa.2024.125866

[36] Pereira R, Morais H, Castro R. Optimising Portugal’s 2050 energy system: Electric vehicles and hydrogen integration using Grey Wolf Optimiser and EnergyPLAN. Energy. 2024;312:133372. doi: 10.1016/j.energy.2024.133372

[37] Pichardo E, Anides E, Vazquez A, Garcia L, Avalos JG, Sánchez G, et al. A compact and high-performance acoustic echo canceller neural processor using grey wolf optimizer along with least mean square algorithms. Mathematics. 2023;11(6):1421. doi: 10.3390/math11061421

[38] Kumar A, Mishra S. A low carbon multi-modal logistics network optimization: A novel neutrosophic mixed integer linear programming approach. J Environ Manage. 2025;387:125924. doi: 10.1016/j.jenvman.2025.125924 40414134

[39] Shi H, Hong Y, Zhang Q, Qin J. Research on cold chain logistics joint distribution vehicle routing optimization based on uncertainty entropy and time-varying network. Entropy. 2025;27(5):540. 10.3390/e27050540 PMC12110850 40422494

[40] Zhang Y, Huang M, Fu Y, Jiang S, Wang X, Fang S-C. Mathematical modeling and optimization of multi-period fourth-party logistics network design problems with customer satisfaction-sensitive demand. Expert Syst Appl. 2025;278:127219. doi: 10.1016/j.eswa.2025.127219

[41] Soria-Arguello I, Villicaña-García E. Logistics optimization applied to redesign operations involving merchandise location, employee ergonomics and distribution network. Mathematics. 2025;13(4):639.

[42] Jiang S, Huang M, Liu Y, Zhang Y, Wang X. Capacity planning to cope with demand surges in fourth-party logistics networks under chance-constrained service levels. Comp Oper Res. 2025;176:106956. doi: 10.1016/j.cor.2024.106956

[43] Niu Y-F, Xiang H-Y, Xu X-Z. Expected performance evaluation and optimization of a multi-distribution multi-state logistics network based on network reliability. Reliability Eng System Safety. 2024;251:110321. doi: 10.1016/j.ress.2024.110321

[44] Wang H, Ran H, Zhang S. Location-routing optimization problem of country-township-village three-level green logistics network considering fuel-electric mixed fleets under carbon emission regulation. Comp Indus Eng. 2024;194:110343. doi: 10.1016/j.cie.2024.110343

[45] He M, Li Q, Wu X, Han X. A novel multi-level reverse logistics network design optimization model for waste batteries considering facility technology types. J Cleaner Prod. 2024;467:142966. doi: 10.1016/j.jclepro.2024.142966

[46] Tosarkani BM, Amin SH, Ghiasvand MR. Designing a sustainable plastic bottle reverse logistics network: A data-driven optimization approach. Expert Syst Appl. 2024;251:123918. doi: 10.1016/j.eswa.2024.123918

[47] Zhu W, Xu B, Guo B, Xie C, Xiong R. Data-efficient and interpretable long-term prognostics of PEMFC degradation via physics-constrained symbolic regression–physics-informed neural network. eTransportation. 2026;29:100615. doi: 10.1016/j.etran.2026.100615

[48] Liu S, Lin R, Jia Y, Wu Q, Chen C. Competitive crack evolution in flawed rocks under thermal–mechanical coupling: A tangential stress and displacement decomposition approach. J Rock Mech Geotech Eng. 2026. doi: 10.1016/j.jrmge.2026.06.006

[49] Zheng L, Li H, Ran L, Li J. Asynchronous distributed learning algorithm for stochastic aggregative games with imperfect information in multi-cluster systems. Neurocomputing. 2027;707:135048. doi: 10.1016/j.neucom.2026.135048

[50] He Y, Xu K, Deng B, Li X, Wu Y, Yang R. On-board adaptive defect detection for wind turbine blade via UAV. Measurement. 2026;123218. doi: 10.1016/j.measurement.2026.123218

[51] Mirjalili S, Mirjalili SM, Lewis A. Grey wolf optimizer. Adv Eng Softw. 2014;69:46–61. doi: 10.1016/j.advengsoft.2013.12.007

[52] Li Y, Shi J, Li C, Zhang K, Xie Y, Zhu L, et al. Synergistic improvement of strength and toughness of zirconia ceramics by introducing zirconia nanofibers. J Alloys Comp. 2026;1056:186592. doi: 10.1016/j.jallcom.2026.186592

[53] Xie J, Jiang Z, Wu Z, Shen J, Zhou Y, Chen Y, et al. Joining of TiAl intermetallic Compound/CoCrFeMnNi high-entropy alloy dissimilar metals by vacuum brazing process: Microstructure, mechanical properties and processing parameters. Intermetallics. 2026;197:109374. doi: 10.1016/j.intermet.2026.109374

[54] Knowles J. ParEGO: a hybrid algorithm with on-line landscape approximation for expensive multiobjective optimization problems. IEEE Trans Evol Computat. 2006;10(1):50–66. doi: 10.1109/tevc.2005.851274

[55] Chugh T, Jin Y, Miettinen K, Hakanen J, Sindhya K. A surrogate-assisted reference vector guided evolutionary algorithm for computationally expensive many-objective optimization. IEEE Trans Evol Computat. 2018;22(1):129–42. doi: 10.1109/tevc.2016.2622301

[56] Gao Y, Zhu H, Zhang Z, Wang Y, Ai Q, Zhu J. Resilient hierarchical control strategy for digital twin cloud-edge collaboration in multi-energy microgrids under non-ideal communication conditions. Appl Energy. 2026;424:128374. doi: 10.1016/j.apenergy.2026.128374

